# The Conundrum of Medical Fracture Prevention in Chronic Kidney Disease—Summary of the Evidence and Pragmatic Clinical Guidance

**DOI:** 10.3390/jcm14228145

**Published:** 2025-11-17

**Authors:** Simeon Schietzel, Uyen Huynh-Do

**Affiliations:** Department of Nephrology and Hypertension, Inselspital, Bern University Hospital, University of Bern, Freiburgstrasse 15, 3010 Bern, Switzerland

**Keywords:** CKD-MBD, kidney, osteoporosis, fracture, BMD

## Abstract

Fragility fractures are a major complication in chronic kidney disease (CKD), yet therapeutic strategies for their prevention remain highly controversial. The unique pathophysiology of CKD–mineral and bone disorder (CKD-MBD), coupled with the paucity of dedicated clinical trials, create substantial uncertainty regarding the efficacy and safety of medical interventions established in the general osteoporosis population. This review summarizes the available evidence regarding fracture risk and bone mineral density including pragmatic clinical guidance for the use of calcium, vitamin D, phosphate binders, calcimimetics, bisphosphonates, denosumab, romosozumab, and teriparatide in patients with advanced non-dialysis CKD, on dialysis, and after kidney transplantation. For calcium, the conflicting balance between skeletal needs and risk of vascular calcification in the setting of declining kidney function and limited evidence for fracture prevention is outlined. For vitamin D, the gap between its widespread clinical use and the inconsistent data on fracture prevention is analyzed including a discussion of target levels in progressive kidney dysfunction. For phosphate binders, the evidence for fracture prevention, showing benefits in dialysis populations, is summarized together with a synthesis of data on potential risks of calcium-based agents. For calcimimetics, the available evidence on their role in fracture prevention, PTH, and calcium control is reviewed. For bisphosphonates, the unresolved question of benefit versus harm in advanced CKD stages are discussed and the evidence regarding efficacy and safety for various clinical settings is disentangled. For denosumab, the current data on fracture prevention is presented with emphasis on its renal-independent pharmacokinetics and strategies to mitigate hypocalcemia and rebound fracture risk. For romosozumab, the promising effects on bone health are reviewed alongside an analysis of cardiovascular safety data. For teriparatide, the limited evidence in patients with low bone turnover disease is evaluated. The review navigates the available evidence and unresolved controversies across therapeutic options, and provides pragmatic guidance to support individualized clinical decision-making.

## 1. Introduction

Medical fracture prevention in chronic kidney disease (CKD) is of high socio-economic relevance, but unfortunately represents a persistent clinical dilemma.

1. Unclear underlying etiology: Even the foundation of any therapeutic approach—i.e., the precise identification of the cause of bone fragility—is often elusive. In aging CKD patients, leading etiologies such as osteoporosis, high and low turnover renal osteodystrophy, osteomalacia, medication effects, frailty, sarcopenia, and recurrent falls with their numerous common risk factors, are all plausible causes. Imaging and laboratory assessments lack diagnostic conclusiveness. Bone histomorphometry could frequently establish a definitive diagnosis thereby enabling treatment paths and targeted management. However, its invasiveness, complex interpretation, and uncertainty regarding effective and safe therapeutic options have led to its underuse in nephrology.
2. Therapeutic complexity: Even when a diagnosis is established (e.g., osteoporosis or adynamic bone disease), treatment remains far from straightforward. Many pharmacologic agents are contraindicated or associated with increased risk in the setting of impaired renal function.
3. Limited and inconsistent evidence: The available clinical data are either sparse—e.g., due to the frequent exclusion of patients with advanced CKD from clinical trials—or extensive but conflicting, as seen with interventions such as vitamin D analogues or calcium-based phosphate binders.
4. Heterogeneous patient populations: Individuals with advanced CKD, those on dialysis, and kidney transplant recipients (KTRs) represent three distinct populations, among whom the extrapolation of study results is only partially valid.

Thus, physicians treating these patients are very often uncertain about which diagnostic pathway resp. and which treatment option to choose, as discussed during the recent Kidney Disease Improving Global Outcomes (KDIGO) controversies conference on CKD–mineral bone disorder [1].

This review therefore aims to bridge the evidence gap by synthesizing current knowledge and unmet needs on pharmacologic fracture prevention across the three populations of advanced CKD, dialysis, and KTRs. On the basis of a precise diagnostic evaluation of the underlying fracture etiology (which is beyond the scope of this article) this work seeks to facilitate pharmacologic treatment decisions within a highly personalized risk–benefit framework. The primary objective of this comprehensive article is therefore to serve as a practical reference to support evidence-based pharmacologic decision-making in response to specific clinical questions.

## 2. Chronic Kidney Disease and Skeletal Health

### Chronic Kidney Disease–Mineral and Bone Disorder

In patients with advanced CKD, a variety of mechanisms contribute to skeletal pathology. Consequently, the umbrella term CKD–mineral and bone disorder (CKD-MBD) has been established (Table 1). Renal osteodystrophy with its high and low turnover forms is the central element of CKD-MBD and the main bone pathology directly attributable to CKD. In addition, osteopenia and osteoporosis are also part of the CKD-MBD spectrum, as low bone mass does not only occur through well-recognized risk factors like age, postmenopausal status, or corticosteroid use, but also as a consequence of renal osteodystrophy. Last and less commonly, osteomalacia is part of CKD-MBD, e.g., as a result of severe vitamin D deficiency. All these conditions may present with varying intensity and degrees of overlap but require fundamentally different treatment approaches. Bone biopsy is the only reliable method for definitive diagnosis and should always be prioritized. All entities of CKD-MBD may lead to reduced bone mass, rendering them indistinguishable from one another on dual-energy X-ray absorptiometry (DEXA). Laboratory and clinical markers may assist in differential assessment, but they cannot reliably replace histological confirmation.

*Renal Osteodystrophy (High and Low Turnover Forms):* Renal osteodystrophy denotes bone disease directly attributable to advanced CKD, characterized by disturbances in turnover, mineralization, trabecular and cortical structure, the lacuno-canalicular network, bone mass, and fibrosis. Its pathogenesis is driven by disordered homeostasis of vitamin D, parathyroid hormone (PTH), calcium, phosphate, acid–base balance, and progressive uremia. Histologically, two principal entities are distinguished according to bone turnover: osteitis fibrosa (high turnover) and adynamic bone disease (low turnover). While bone biopsy remains the diagnostic gold standard, circulating markers of bone metabolism (e.g., PTH, bone-specific and total alkaline phosphatase, P1NP, TRAP5b, calcium, and phosphate) may support clinical assessment, albeit with limited specificity due to substantial overlap between phenotypes (Table 2). Adynamic bone disease is increasingly recognized as an essential form of renal osteodystrophy, surpassing osteitis fibrosa in prevalence and in its adverse effects on bone and cardiovascular (CV) health. Risk factors include advanced age, diabetes, suppression or surgical removal of the parathyroid glands, excessive calcium, phosphate, or vitamin D exposure, calcitriol deficiency, use of calcimimetics, high comorbidity burden, malnutrition, systemic inflammation, oxidative stress, accumulation of PTH fragments and uremic toxins, and treatment with antiresorptive agents such as bisphosphonates or denosumab.

*Osteopenia and Osteoporosis*: As in the general population, patients with CKD are subject to conventional risk factors for reduced bone mass, including, amongst others, older age, female sex, postmenopausal status, family history, lifestyle factors, and medication use. However, in CKD, reduced bone mass may also arise from renal osteodystrophy (high or low turnover) or from osteomalacia, e.g., due to severe vitamin D deficiency. Measurement of bone mineral density (BMD) can diagnose osteopenia or osteoporosis but provide no information regarding the underlying etiology, which may differ fundamentally. In age-related osteoporosis, bone loss primarily results from an imbalance between resorption and formation, leading to thinning and loss of trabeculae and increased cortical porosity, while mineralization, fibrosis, osteoid volume, and turnover are only modestly impaired. By contrast, in renal osteodystrophy, bone metabolism, and structure are profoundly disrupted at multiple levels. Bone biopsy remains the only reliable method to establish the prevailing etiology. The substantial histomorphometric differences described underscore the therapeutic uncertainty as to whether, in renal osteodystrophy, increasing bone mass (e.g., via antiresorptive therapy) enhances mechanical competence, or merely increases density within an intrinsically compromised skeletal structure.

*Osteomalacia*: Due to increased awareness and routine correction of vitamin D deficiency, the incidence of overt osteomalacia has decreased; however, considering this condition in the context of bone fragility remains important. In CKD, impaired activation of vitamin D may result in insufficient biological activity despite adequate 25 hydroxy vitamin D (25[OH]D) levels, thereby contributing to mineralization deficits. Definitive diagnosis and quantification remain possible, however, only by histological assessment.

## 3. Calcium

### 3.1. Rationale and Guidelines

The rationale for supplementation of elementary calcium in osteoporosis lies in its essential role for bone mineralization and the maintenance of normal bone metabolism.

Recommendations from recognized professional societies regarding daily intake of elementary calcium for the general population range from 700 mg (National Health Service, UK [8]) to 1000 mg (D-A-CH, German–Austrian–Swiss Nutrition Societies [9]) and up to 1200 mg (women > 50/men > 70 years: Health and Medicine Division of the National Academies of Sciences, Engineering, and Medicine [HMD/NASEM]—the former Institute of Medicine [10]).

In the context of osteoporosis or increased fracture risk, recommendations vary between 700 mg (UK, National Osteoporosis Guideline Group [11]), 1000 mg (Swiss Association against Osteoporosis [12], German Society for Osteology [13]; women 19–50/men 19–70 years: Bone Health & Osteoporosis Foundation [BHOF] [14], International Osteoporosis Foundation [IOF] [15], Osteoporosis Canada [OC] [16]), 1200 mg (women > 50/men > 70 years: BHOF [14], HMD/NASEM [10], IOF [15], OC [16]; women > 50 years: American Association of Clinical Endocrinologists/American College of Endocrinology Clinical Practice Guidelines [17]), and up to 1300 mg (Royal Australian College of General Practitioners [18]).

Supplementation may be considered in cases of clearly inadequate intake or in the context of osteoporosis therapy. There is a general consensus that increasing calcium intake through diet is always preferable to supplementation. It is important to be aware that calcium supplementation alone does not reduce fracture incidence in older adults or in patients with osteoporosis; in some studies, a benefit has been demonstrated in combination with vitamin D in certain subgroups (e.g., older adults). Calcium intakes above 2000 mg/day are generally regarded as unfavorable.

With regard to CKD, a European consensus statement recommends 800–1000 mg of elementary calcium per day not exceeding 1500 mg per day [19]. Recognized recommendations for a daily intake of elementary calcium to maintain a neutral calcium balance according to kidney function are as follows [20]:

- Normal kidney function: 1000–1300 mg (adjusted for age)
- CKD stages 3–4: 800–1000 mg
- CKD stage 5 non-dialysis: 800–1000 mg or less
- Hemodialysis: No exact amounts provided; adjust individually, aiming for the lower range

It is crucial to be aware that overall calcium balance in patients with advanced CKD is positive. This is true despite the classic finding of hypocalcemia, which disguises the fact that reduced calcium excretion, progressively autonomous PTH secretion, and hyperphosphatemia constantly mediate mobilization and storage of excess calcium in non-bone tissues, contributing to the overriding CV risk profile of this population. Looking at a patient’s calcium intake, all sources need to be taken into account, such as dietary intake, supplements, and phosphate binders. Calcium-based phosphate binders (CBPB) contain high amounts of elementary calcium (calcium acetate = 25% elementary calcium and calcium carbonate = 40% elementary calcium). The KDIGO CKD-MBD guidelines recommend that mild hypocalcemia may be acceptable, that hypercalcemia is to be avoided, and that the use of CBPB be limited. In dialysis patients, calcium intake should be adjusted to individual co-medication (e.g., vitamin D and calcimimetics) to avoid calcium overload (opinion-based). While the evidence is limited, dialysate calcium concentration should be low (1.25–1.50 mmol/L) [2]. After kidney transplantation, uremia subsides, allograft function readily activates 25(OH)D, and persistent parathyroid autonomy meets resolving PTH resistance of bone stores resulting in transient or persistent hypercalcemia. Management comprises reduction in excess calcium or vitamin D intake as well as calcimimetics; parathyroidectomy in prolonged, refractory cases. Pronounced and/or prolonged hypercalcemia harbors the risk of significant tissue calcification affecting CV prognosis and potentially allograft function as well.

### 3.2. Evidence on Fracture Prevention and Bone Mineral Density

**Advanced CKD:**

*Fractures:* We are not aware of clinical trials primarily investigating the effect of calcium intake on fracture prevention or BMD in the advanced CKD population. Available data are derived from randomized controlled trials (RCTs) comparing calcium based with calcium-free phosphate binders. A 2018 meta-analysis including 1467 participants with CKD 2–5 (non-dialysis), comparing CBPB and non-CBPB to placebo (15 studies) or usual care (25 studies) found no clinically important benefits of any phosphate binder on fractures [21]. While a solid body of evidence advocates against a positive calcium balance accepting mild hypocalcemia, and recent analyses of data from the Chronic Renal Insufficiency Cohort (CRIC; n = 3939) showed a significant association between time-updated hypocalcemia exposure and increased fractures: aHR 1.44 (95% CI 1.02–2.03) per 0.25 mmol/L calcium < 2.13 mmol/L [22].

*BMD:* We are not aware of studies investigating the effect of calcium on BMD in non-dialysis CKD.

**Dialysis:**

*Fractures:* In short, no effect of serum calcium or phosphate binder-based calcium intake on fracture prevention has been found. A recent meta-analysis found a trend but no significant effect of hypercalcemia (eight studies, RR 0.90 [95% CI, 0.77–1.05], *p* = 0.188, I^2^ = 67.1%) as well as hypocalcemia (five studies, RR 1.11 [95% CI, 0.99–1.24], *p* = 0.087, I^2^ = 56%) on fracture risk [23]. A meta-analysis of RCTs did not find a clinically important benefit of any phosphate binder (CBPB or non-CBPB) on fracture risk [21]. A large Japanese RCT (273 dialysis facilities) found no difference in hip fractures in patients receiving lanthanum (n = 1154) or calcium carbonate (n = 1155) over a median follow-up of 3.2 years (HR 1.21 [95% CI, 0.62–2.35]; *p* = 0.58) [24]. A smaller RCT in 43 diabetes patients found no significant difference in fracture rates with lanthanum versus (vs.) calcium carbonate within 24 months follow-up (0/16 vs. 3/22, *p* = 0.25) [25].

*BMD:* The evidence is very limited with inconsistent findings. Two out of three very small RCTs (n = 43, n = 45) did not find BMD differences with lanthanum vs. calcium carbonate over 24 and 18 months, respectively [25,26]. One RCT (n = 18) investigated high doses of elemental calcium intake (2 g/d) vs. placebo and found bone mineral content differences of 10.2% (*p* < 0.05) after 6 months follow-up [27]. A 4 years retrospective analysis showed an independent effect of CBPB on BMD maintenance compared to non-CBPB use (β-coefficient 0.033 [95%CI 0.001–0.065]; *p* = 0.046) [28]. In a cross-sectional Japanese study of 321 dialysis patients, the only medication associated with lower osteoporosis risk were CBPBs (OR 0.41 [95% CI, 0.21–0.81]; *p* = 0.011) with particular risk reductions within subgroups of dialysis vintage ≥ 10 years, albumin below 35 g/L, active vitamin D, and no proton pump inhibitor use [29].

**Kidney transplant:**

*Fractures and BMD:* Fracture risk is markedly elevated; however, data on the effect of calcium supplementation on fracture prevention or BMD is lacking.

### 3.3. Safety of Calcium Supplementation

CKD is among the most severe CV risk factors [30]. The current KDIGO guidelines emphasize the paramount importance of stratifying and managing CV risk in patients with CKD. Likewise, the European Society of Cardiology and the European Atherosclerosis Society (ESC/EAS) recently classified moderate and severe CKD-stages as belonging to the high and very high CV risk categories [31]. CKD-MBD plays a central pathophysiological role in CV risk, as positive calcium and phosphate balances and secondary hyperparathyroidism—together with oxidative stress, chronic inflammation, uremia, and exposure to dialysis treatments—are all independently associated with vascular calcification [32]. In a cross-sectional study involving 205 dialysis patients, an increase of 0.25 mmol/L in serum calcium was associated with a degree of vascular calcification equivalent to that observed after >5 years of dialysis vintage [33]. Most of the safety data in CKD originates from comparisons between the use of CBPB versus non-CBPB. In a recent European consensus statement, the literature on the risks of CBPB by dose of elementary calcium has thoroughly been investigated [34] as follows:

*Vascular calcification:* Trials comparing CBPB versus non-CBPB (sevelamer and lanthanum) found mixed results, however, with the majority of studies showing greater progression of vascular calcification with CBPB (overall ratio 3:2 studies in CKD, 7:3 studies in dialysis).

*Mortality and CV events:* Only one study investigated a pure CKD population (CKD 3–5) without including dialysis patients. There was no clear conclusion on the risk of all-cause mortality or CV events with regard to the amount of calcium intake. Looking at studies that include dialysis patients (CKD 3–5D), four meta-analyses (15–77 trials) and a Cochrane review (104 trials) provide safety data comparing CBPB vs. non-CBPB. All of these five investigations found a reduced risk of all-cause mortality with non-CBPB (especially sevelamer). One meta-analysis and the Cochrane review investigated CV mortality showing mixed results with no difference and reduced risk, respectively, in favor of non-CBPB. In dialysis patients only, the COSMOS study (2015) found a significant U-shaped association between serum calcium and mortality. In addition, mortality decreased when high calcium levels were actively lowered [35].

### 3.4. Pragmatic Clinical Approach

Target a calcium intake compatible with a neutral calcium balance adjusted to kidney function (CKD 3–4: 800–1000 mg, CKD five non-dialysis: 800–1000 mg or less, dialysis: adjust individually, aiming for the lower range). For the evaluation of calcium intake, take dietary sources, supplementation, and CBPB into account. Accept mild hypocalcemia; avoid sustained hypercalcemia and marked hypocalcemia. Avoid increased calcium doses for fracture or BMD endpoints, as evidence does not support clear benefit. Tailor use of calcium-based phosphate binders to individual risk–benefit profiles within the broader CKD-MBD management strategy. When targeting a higher calcium intake or to manage pronounced deficiencies, dietary sources should be prioritized over supplementation whenever feasible.

## 4. Vitamin D

### 4.1. Rationale and Guidelines

Vitamin D is essential for skeletal health through its role in intestinal calcium absorption, bone mineralization, regulation of phosphate metabolism, and modulation of osteoblast and osteoclast activity. It protects against osteomalacia, osteoporosis, and secondary hyperparathyroidism. Serum 25(OH)D status depends on multiple factors and varies across populations. Deficiency is highly prevalent both in the general population and among patients with CKD. A recent meta-analysis including data from 586 studies and 2,360,136 participants without kidney disease reported a pooled global prevalence of 25(OH)D deficiency (<30 nmol/L) of 18% and insufficiency (<50 nmol/L) of 47% [36]. With progressive renal impairment, 25(OH)D deficiency often becomes even more common, with insufficiency (<50 nmol/L) reported in up to 86% of patients with advanced CKD stages [32,33]. A key driver of CKD–MBD is, however, the progressive loss of renal 1α-hydroxylase activity, resulting in inadequate 1,25(OH)_2_D synthesis even with sufficient 25(OH)D levels. While 1,25(OH)D deficiency (<43 pmol/L) is reported to occur in about 3.1% of individuals with normal kidney function, its prevalence has been shown to rise considerably with declining kidney function—each 10 mL/min/1.73 m^2^ decrease in GFR increasing the odds by 1.86 (95% CI, 1.64–2.09)—with median levels of 32.6 (22.6–45.4) pmol/L in CKD stage 5 [34,35]. KDIGO CKD-MBD guidelines recommend correction of 25(OH)D deficiency in all advanced CKD stages (including dialysis and transplantation), with treatment strategies and targets aligned with those for the general population. Optimal thresholds for sufficiency remain debated. The National Academy of Medicine, the National Osteoporosis Foundation, the American Geriatrics Society, and the U.S. Preventive Services Task Force define sufficiency as >50 nmol/L, which meets the requirements of 97.5% of the general population. The National Academy of Medicine further advises daily intakes of 600 IU vitamin D for individuals aged 1–70 years and 800 IU for those ≥71 years to achieve this level. In contrast, the Endocrine Society, the American Association of Clinical Endocrinologists, and the Italian Association of Clinical Endocrinologists recommend >75 nmol/L, referring to the association between 25(OH)D level and bone health, lower risk of osteomalacia and rickets, and particular benefits for older adults and patients at high fracture risk. Of note, units tend to cause confusion; divide existing recommendations of >50 nmol/L and >75 nmol/L by 2.5 to get recommendations in ng/mL (>20 ng/mL and >40 ng/mL). In advanced CKD, sufficiency of 25(OH)D alone is not enough, as conversion to active vitamin D is impaired with parathyroid autonomy and uremia predominating. Consequently, vitamin D supplementation strategies—including dosing intensity and the use of active analogues—is increasingly guided by PTH, calcium, and phosphate levels, as recommended in the 2017 KDIGO CKD-MBD guidelines [2].

At this point, discussing management of PTH levels seems beneficial. PTH is influenced by multiple factors (vitamin D status, calcium and phosphate balance, renal function, degree of uremia, acid–base status, age, etc.). Mild secondary hyperparathyroidism may appear early as GFR declines (e.g., 59% in eGFR < 60 mL/min/1.73 m^2^ [37]) but typically exacerbates only in advanced CKD [38]. Studies in CKD patients without vitamin D substitution report PTH levels of 36 (IQR 25, 49) pg/mL or 26.4 (SD 28.11) pg/mL at GFR > 60mL/min/1.73 m^2^ with up to 185.5 (SD 159.88) pg/mL and 184 (IQR 109, 304) pg/mL at GFR < 30 and <15 mL/min/1.73 m^2^, respectively [35,36]. Across all CKD stages—and most prominently in severe CKD—PTH levels are dependent on vitamin D status. Moderate secondary hyperparathyroidism represents a physiological adaptation to maintain bone metabolism as renal function declines. In advanced and pre-dialysis CKD, however, PTH levels may rise sharply [38]. At this stage, it remains uncertain whether this increase reflects an appropriate adaptive response preserving bone and electrolyte homeostasis, or rather a maladaptive over-secretion due to progressive parathyroid autonomy. No universally accepted PTH targets exist [2]. Ultimately, potential triggers of secondary hyperparathyroidism should be identified and corrected where feasible and without competing risks. Thereafter, level and temporal trends of PTH—together with CKD duration, bone turnover markers, and PTH reactivity—should guide assessment of “PTH-adequacy”. This allows an educated judgement as to whether the level of PTH elevation is appropriate, too high (risk of high-turnover bone disease), or too low (risk of ABD). In pre-dialysis CKD, a gradual alignment toward PTH targets suggested in hemodialysis (Table 2) appears reasonable. However, target ranges are broad and specific supportive data are missing.

### 4.2. Evidence on Fracture Prevention and Bone Mineral Density

**Advanced CKD:**

*Fractures:* The limited available evidence provides no signal that either active or non-active vitamin D reduces fracture incidence in patients with advanced CKD. While the body of evidence appears substantial—comprising three recent meta-analyses [23,39,40], two secondary RCT analyses (CREDENCE, PRIMO) [41,42], two large observational studies [22,43], and follow-up periods mostly exceeding 12 months—a closer examination reveals that the question remains only partially addressed.

The three meta-analyses ultimately draw on a total of only six studies relevant to advanced CKD (two small RCTs, one secondary RCT analysis, one cross-sectional study, and three studies without specified design). Analyses still include participants with only moderate CKD stages (Liu: CKD 2–3; Yeoung and Khalifi: CKD 3–5), are based on very few fracture events, and often omit critical details such as the type of vitamin D administered (active, non-active, or both). Similarly, the post hoc RCT analyses (CREDENCE trial, n = 4937 [41]; PRIMO trial, n = 227 [42]) and the observational studies (CRIC cohort, n = 3939 [22]; cross-sectional study, n = 17,608 [43]) allow only limited conclusions for patients with advanced CKD, as they predominantly included broader CKD stages (CKD 2–3, GFR 15–60 and 20–70 mL/min/1.73 m^2^, CKD 2–3, respectively). Notwithstanding these limitations, none of the cited studies demonstrated a significant reduction in fracture risk with vitamin D. Only the secondary analysis of the CREDENCE trial, with its large patient population and robust fracture ascertainment (159 fractures, recorded as a key safety outcome), reported a statistically significant result—namely, an increased fracture risk associated with vitamin D. However, the authors interpreted this finding as the result of indication bias [41].

*BMD:* The available evidence is scarce and without focus on advanced CKD stages; however, limited BMD gains upon treatment with vitamin D have been found. A small but double-blind RCT (n = 36) compared an 18 month treatment with alfacalcidol (1(OH) vitamin D, which is non-active but independent from renal activation) with placebo and found BMD gains of 3% (total femur), 4.2% (spine), and 4.9% (femoral neck). Participants had a mean eGFR of 49 (20) mL/min/1.73 m^2^ [44]. A post hoc analysis of the DECALYOS II trial (n = 322) showed significantly reduced BMD losses at the distal radius site comparing cholecalciferol (vitamin D3, which is non-active and requires activation by liver and kidney) 800 U/d plus calcium 1200 mg/d with placebo. Effects after 24 months’ follow-up were independent of baseline (BL) kidney function (eGFR < 45 up to >60 mL/min/1.73 m^2^) but tiny, questioning clinical significance (e.g., −0.01 vs. −0.03 g/cm^3^ for eGFR < 45 mL/min/1.73 m^2^) [45]. A small prospective controlled trial (n = 25) in CKD patients with eGFR < 50 mL/min/1.73 m^2^ reported small BMD gains of 0.028 g/cm^2^ (lumbar spine) and 0.035 g/cm^2^ (femoral neck) comparing 1 year treatment of calcitriol 0.25 ug/d with placebo (*p* < 0.01) [46].

**Dialysis:**

*Fractures:* The most important study in this context—the Dialysis Outcomes and Practice Patterns Study (DOPPS)—included over 40,000 dialysis patients across 21 countries and found no effect of active vitamin D on the primary outcome of fracture risk (any fracture: aHR 1.02 [95% CI, 0.90–1.17], and hip fracture: aHR 1.00 [95% CI, 0.81–1.23]). Results were consistently negative regardless of crude or meticulous adjustment, higher versus lower dosing, or oral versus intravenous administration [47].

With regard to clinical trial evidence level, we are not aware of studies investigating the effect of vitamin D on fractures as the primary objective within the dialysis population. Valuable indirect RCT evidence comes from the large EVOLVE trial (n = 3883) that investigated the effect of cinacalcet versus placebo on mortality and CV events. Secondary analyses of fractures with regard to concomitant use of active vitamin D (cinacalcet +/− paricalcitol vs. placebo) did not show a significant difference in fracture incidence [48].

Five clinical trials reported fractures as part of their adverse events documentation without further analyses (3× HD [49,50,51], n = 62, n = 76, n = 964; 2× PD [52,53], n = 65, n = 90). Fracture rates were very similar between active vitamin D and placebo groups but low event rates make valid inference challenging (e.g., 0/34 vs. 1/28 or 1/34 vs. 2/31). The largest of these trials (n = 964) compared alfacalcidol (0.5 µg/d) to placebo and found no difference in the proportion of patients with AEs of falls and/or fractures within 2 years (9/488 vs. 12/476, RR 0.73 [95% CI, 0.31–1.72]) [51]. Baker et al. provided data for the longest follow-up time (5 years), documenting the same number of fractures (1/38 vs. 1/38) with calcitriol (0.25 µg to 1 µg/d) versus placebo. However, included participants were without biochemical or radiological evidence of bone disease at BL [50].

In contrast to the negative evidence provided by DOPSS and secondary RCT analyses cited above, four observational studies with fractures as primary objectives found positive results. Two retrospective cohort studies (Scottland, n = 907; Portugal, n = 341) found active vitamin D use being associated with reduced fracture risk (HR 0.54 [95% CI, 0.34–0.85]; *p* = 0.008 and HR 0.89 [95% CI, 0.86–0.95]; *p* = 0.03, respectively) [54,55]. A cross-sectional study from Italy (n = 387) found an association between oral calcitriol intake and fracture prevalence (OR 0.6 [95% CI, 0.36–0.99]; *p* = 0.043) [56]. Japanese registry data (n = 208,512) showed a significant association between active vitamin D intake and lower incidence of hip fractures, however, only among subjects with good, but not with poor performance status [57]. Low serum vitamin D levels were not associated with fracture risk in a small prospective cohort from Czech Republic (n = 59) [58].

Three meta-analyses include subgroups of dialysis patients mainly comprising the primary evidence cited above. Fracture rates from RCTs adverse events with mainly very low numbers of fracture were not different compared to placebo (Khelifi, 2022 [40]: four trials, n = 1254, active vitamin D versus placebo or control, RR 0.88 [95% CI, 0.40–1.96], I2 = 0%. Yeung, 2023 [39]: five trials, n = 1220, presumably active and non-active vitamin D combined versus placebo: RR 0.68 [95% CI, 0.29–1.59]). Liu et al. showed a positive effect summarizing five studies that compared active vitamin D with placebo (RR 0.82 [95% CI, 0.74–0.92], I^2^ = 75.8%) [23]. However, the primary objective (association of aberrant metabolic markers with fracture risk) resulted in inclusion of the EVOLVE vitamin D group and the above-mentioned positive observational evidence excluding several studies with negative results. Of note, none of the meta-analyses included results from the DOPPS study.

With regard to fracture prevention by type of vitamin D (active versus non-active), available evidence is insufficient to draw meaningful conclusions. Yeung et al. investigated dialysis and CKD patients combined and found no significant differences comparing non-active (three trials), active (five trials) or both (eight trials) with placebo (RR 0.46 [95% CI, 0.01–21.32], RR 0.74 [95% CI, 0.42–1.28], RR 0.68 [95% CI, 0.37–1.23]) [39]. Positive observational evidence comes from studies investigating active vitamin D.

With regard to concomitant calcium substitution, indirect data come from the DOPPS study, where use versus non-use of calcium-containing phosphate binders did not change the null-effect of active vitamin D on fracture risk [47].

*BMD:* We are not aware of significant evidence regarding vitamin D and BMD in dialysis patients.

**Kidney transplant:**

*Fractures:* For fracture prevention with vitamin D in KTRs, data from one large RCT is available. The multicenter, double-blind VITALE RCT [59] (n = 536) investigated high- versus low-dose cholecalciferol starting 12 to 48 months after transplantation with a treatment duration of 22 months. Treatment protocol was 100,000 IU vs. 12,000 IU every 2 weeks for two months and monthly thereafter, translating to equivalent daily doses of 6600 IU vs. 800 IU for two months followed by 3300 IU vs. 400 IU thereafter. Difference in symptomatic fractures were n = 3 (1%) for high dose vs. n = 12 (4%) for low dose vitamin D (HR 0.24 [95% CI, 0.07–0.86]; *p* = 0.03). Median daily calcium intake was 758 mg [584, 979], 60% of participants took oral corticosteroids at BL, 15% had a history of fractures, and median dialysis vintage was 21.0 months [12.0, 39.5]. Twenty-four months serum 25(OH)D levels were 107.5 (32.1) versus 62.7 (18.4) nmol/L (*p* < 0.0001) and serum iPTH levels 90.15 (80.32) versus 93.25 (66.24) pg/mL (*p* = 0.002). Adverse events did not differ between groups. Remarkably, serum calcium and serum phosphate level also did not differ between groups. However, in the per protocol analysis, the incidence of a urine calcium-to-creatinine ratio above 0.375, was significantly higher in the high-dose group [59]. We are not aware of studies investigating active vitamin D against placebo regarding fracture outcome.

*BMD:* We are not aware of significant evidence regarding vitamin D and BMD in KTRs.

### 4.3. Safety of Vitamin D Supplementation

Vitamin D supplementation is generally well tolerated, with hypercalcemia representing the principal potential adverse effect. A recent meta-analysis of RCTs in non-dialysis CKD patients (12 trials, 1537 participants) demonstrated a significantly increased risk of hypercalcemia with vitamin D compared to placebo (RR 2.63 [95% CI, 1.29–5.38]) [39]. Notably, this analysis did not differentiate between active and non-active vitamin D preparations, which were presumably pooled. In the combined non-dialysis and dialysis population, only active vitamin D preparations increased the risk of hypercalcemia (active vitamin D: 17 trials, RR 2.62 [95% CI, 1.76–3.90]; I^2^ = 0%, non-active vitamin D: 11 trials, RR 0.68 [95% CI, 0.39–1.19]; I^2^ = 0%). In contrast, with active and non-active preparations combined, the risk was significantly elevated (RR 1.75 [95% CI, 1.13–2.72]; I^2^ = 26%), although this was not observed in trials restricted to patients with secondary hyperparathyroidism (10 trials, n = 2442, RR 2.18 [95% CI, 0.85–5.55]). Importantly, no increased risk of hyperphosphatemia was observed across the meta-analysis, either overall (RR 0.96 [95% CI, 0.75–1.22]) or for active versus non-active vitamin D. In summary, the risk of hypercalcemia with vitamin D therapy in CKD is robustly demonstrated—particularly with active preparations, though less so with non-active compounds. The individual threshold at which hypercalcemia emerges depends on multiple factors, including kidney function, calcium intake, PTH levels, and bone metabolic turnover capacity. Vitamin D remains an essential component in the management of CKD-MBD, but appropriate selection of preparation and dose requires careful individualization, with vigilant monitoring to avoid hypercalcemia. With regard to hard outcomes, a large multicenter Japanese RCT (n = 964) in dialysis patients reported no significant effect of alfacalcidol (0.5 μg/day) compared with placebo over 48 months on either CV events (HR 1.25; 95% CI, 0.94–1.67; *p* = 0.13) or all-cause mortality (HR 1.12; 95% CI, 0.83–1.52; *p* = 0.46) [51].

### 4.4. Pragmatic Clinical Approach

In advanced CKD, there is no evidence that vitamin D reduces fractures although representation of respective patients in clinical studies is limited. Evidence regarding BMD is minimal, with only very small studies suggesting limited benefits. Aim for a 25(OH)D level of >50 nmol/L, adjusting to >75 nmol/L in increased fracture risk or if further lowing of PTH is desired. PTH target levels in advanced CKD are unknown. Between advanced CKD and end-stage kidney disease (ESKD), PTH levels typically rise from moderately elevated to progressively approaching the target ranges commonly applied in dialysis (2–9 times the upper limit of normal [ULN]). The challenge in advanced CKD stages is to take an educated guess for an individualized PTH target that prevents overtly high turnover but avoids over suppression, enabling a “healthy”, responsive bone turnover.

In dialysis, evidence from DOPPS, EVOLVE, and smaller trials shows no effect, while observational cohorts suggest potential fracture benefits of active vitamin D. No benefit has been shown for combinations with calcium-based phosphate binders. Data on BMD is missing. In ESKD, 25(OH)D levels are not reliable markers of systemic vitamin D effects anymore as renal conversion capacity is partially or completely lost. Guide non-active and active vitamin D therapy by individual CKD-MBD parameters (PTH, Ph, AP, Ca, and bone-specific AP), enable adequate bone turnover without over suppression of PTH with risks of ABD. Avoid hypercalcemia, taking the intake of vitamin D, calcium, and calcium-based phosphate binders into account.

In KTRs, data are almost absent, though one large trial indicated significant fracture reduction with high-dose non-active vitamin D. In KTRs, risk of hypercalcemia is increased.

Overall, vitamin D supplementation is safe, but hypercalcemia—especially with active vitamin D—is the limiting factor that needs to be monitored for and avoided. In hypercalcemia, restriction of vitamin D, avoidance of positive calcium balance, and use of calcimimetics guide clinical management.

## 5. Phosphate Binders

### 5.1. Rationale and Guidelines

With advancing CKD, progressive hyperphosphatemia constitutes a central and direct burden on skeletal health. The underlying pathophysiological mechanisms include stimulation of Fibroblast Growth Factor (FGF23) and PTH, suppression of 1α-hydroxylase, and direct cellular effects resulting in secondary hyperparathyroidism, impaired mineralization, enhanced bone resorption, and disturbed skeletal homeostasis. Current guidelines emphasize the importance of phosphate control across all advanced stages of the CKD spectrum, incorporating dietary measures, pharmacological phosphate binding with consideration of overall calcium balance, adjustment of dialysis dose, and awareness of hypophosphatemia risk in KTRs. Target ranges remain a matter of debate; however, aiming toward values within the normal range is generally recommended for both non-dialysis and dialysis CKD patients [2]. KDIGO refers to evidence not justifying phosphate-lowering therapy in patients with normal serum phosphate concentrations [2]. The Kidney Disease Outcomes Quality Initiative (KDOQI) emphasizes that normal reference ranges for serum phosphate are not clearly defined, that the current evidence base remains insufficient, and that, particularly in cases of increasing phosphate dynamics even within the normal range, dietary phosphate restriction should not generally be dismissed as ineffective but may, in selected situations, help counteract the accelerated development of secondary hyperparathyroidism [6].

### 5.2. Evidence on Fracture Prevention and Bone Mineral Density

**Advanced CKD:**

*Fractures:* Although still a matter of debate, hyperphosphatemia is likely to increase fracture risk in both the general population and in patients with CKD. For the general population, a pooled analysis from the Rotterdam Study (RS; n = 6791) and the U.S. Osteoporotic Fractures in Men study (MrOS; n = 5425), with follow-up periods between 6.6 to 10.9 years, demonstrated a HR for incident fracture of 1.47 (95% CI, 1.31–1.65); *p* < 0.001, per 0.32 mmol/L rise in serum phosphate. This association was independent of 25(OH)D, PTH, FGF23, dietary phosphate intake, kidney function, and bone turnover markers, and was accompanied by a significant dose–response relationship. Notably, participants had phosphate levels within the normal range (0.81–1.45 mmol/L). A potential threshold effect was suggested, with fracture risk increasing above ~1.1 mmol/L in men and ~1.2 mmol/L in women, although trend analyses indicate that risk may begin to rise at even lower levels. For those with eGFR < 58 mL/min/1.73 m^2^, the association was particularly pronounced in men, with hyperphosphatemia conferring a nearly twofold increase in fracture risk (HR 1.93 [95% CI, 1.42–2.62]) [60]. With regard to the CKD population, a recent meta-analysis of RCTs and quasi-RCTs investigating the association of aberrant metabolic marker with fracture risk found only one study in non-dialysis CKD [23]. In 126 patients with diabetes and CKD 2–3, phosphate > 1.78 mmol/L was not associated with increased fracture risk (RR 1.06 [95% CI, 0.85–4.59]; *p* = 0.97) [61]. We are not aware of data regarding hypophosphatemia and fracture risk.

With respect to phosphate binders and fracture risk, a recent meta-analysis in CKD found no significant association (RR 1.07 [95% CI, 0.90–1.27]) [23]. The three studies included were observational, comprised CKD stages 2, 3, and 1–5, respectively, and had follow-up periods of 70 months and 5 years (on cross-sectional study) [43,62,63]. Similarly, a Cochrane review of randomized and quasi-randomized trials found three studies in non-dialysis CKD patients reporting no significant effect of phosphate binders on fracture outcomes [64]: Specifically, lanthanum vs. placebo/usual care (CKD 2–5; 1/28 vs. 2/57; RR 1.02 [95% CI, 0.10–10.75]), sevelamer vs. placebo/usual care (CKD 2–5; 0/30 vs. 2/57; RR 0.37 [95% CI, 0.02–7.55], and 1/101 vs. 0/101; RR 3.00 [95% CI, 0.12–72.78]), sevelamer vs. calcium acetate (CKD 2–5; 2/30 vs. 1/30; RR 2.00 [95% CI, 0.19–20.90]), and sevelamer vs. lanthanum (CKD 2–5; 2/30 vs. 1/28; RR 1.87 [95% CI, 0.18–19.47]). In combined populations of non-dialysis and dialysis patients (two studies), findings were likewise null: iron-based binders vs. placebo/usual care (CKD G2–5D; 2/75 vs. 1/73; RR 1.95 [95% CI, 0.18–21.01]) and lanthanum vs. calcium carbonate (CKD 2–5D; 19/1063 vs. 16/1072; RR 1.20 [95% CI, 0.62–2.32]) [64].

BMD: We are not aware of studies investigating the effect of phosphate binders on BMD in non-dialysis CKD patients.

**Dialysis:** Regarding phosphate level and fracture risk, a recent meta-analysis found phosphate levels > 1.78 mmol/L (normal range defined as 1.13–1.78 mmol/L according to K/DOQI) associated with increased fracture risk (RR 1.08; 95% CI, 1.02–1.15; *p* = 0.013, I^2^ = 2.8%; 8/9 individual studies did not reach significance). Importantly, hypophosphatemia (levels < 1.13 mmol/L) were likewise associated with increased fracture risk (RR 1.13 [95% CI, 1.02–1.25]; *p* = 0.022; 5/5 individual studies non-significant) [23]. The COSMOS study, a prospective cohort analysis including 6797 dialysis patients across 20 European countries with a median follow-up of 24 months, reported that baseline serum phosphate > 1.97 mmol/L compared to the reference of 1.39–1.97 mmol/L was associated with higher risk for fragility fractures (subHR 1.42 [95% CI, 1.02–1.98]). The association was not sustained when phosphate was analyzed as a continuous variable [65]. An earlier large retrospective study comprising 40,538 dialysis patients found serum phosphorus to be significantly related to fracture-related hospitalization (n = 257), with a RR of 1.12 (95% CI, 1.03–1.22) per 0.32 mmol/L increase in serum phosphorus [66].

Fracture: Available evidence comprises one meta-analysis [23] and two database studies [63,67] providing solid evidence for fracture prevention with phosphate binding, whereas two Cochrane reviews of RCTs [21,64] yielded mixed results.

Meta-analytic evidence is very robust (two RCTs, follow-up 3 und 5 years; five cohort studies, follow-up 2–10 years, one cross-sectional study) showing a 21% reduced fracture incidence with the use of phosphate binders: RR 0.79 (95% CI, 0.70–0.89), I^2^ = 48.8% [23]. Evidence based on insurance databases [63,67] (Kim et al. and Kwon et al.) is of lower scientific value; however, the number of participants is huge (n = 69.368, 8.3% PD and n = 13427, 9.9% PD) and follow-up times are adequate (36.0 [IQR 15.8, 65.1] months and 4 years). Kim et al. found aHRs for overall fracture risk reduction of 0.76 (0.62–0.94); *p* = 0.011 (0.85 [0.8–0.9]; *p* < 0.001 with CBPB and 0.85 [0.72–0.99]; *p* = 0.38 with non-CBPB). A post hoc analysis revealed no significant difference in the risk of total, vertebral, and non-vertebral fractures according to binder type. Fracture risk reduction increased time-dependence with larger aHRs with <3, 3–6, or >6 months of binder use [67]. Kwon et al. found that not using a phosphate binder increased fracture risk by 20% within 4 years (aHR 1.20 [95% CI 1.09–1.31]; *p* < 0.001) [63]. The Cochrane reviews by Natale et al. and Ruospo et al. provided evidence from three RCTs, of which, two found no effect on fracture risk (sevelamer vs. iron-based binders: RR 0.65 [95% CI, 0.03–15.80] and lanthanum carbonate versus sucroferric oxyhydroxide: RR 1.00 [95% CI, 0.14–6.92]), whereas one trial did find a benefit (sevelamer vs. bixalomer: RR 0.33 [95% CI, 0.01–8.01]. However, event rates (0/68 vs. 1/134, 2/78 vs. 2/78 and 0/55 vs. 1/55) and certainty of evidence was very low [21,64].

BMD: Evidence is very scarce (one cross-sectional study, one small retrospective cohort study) but may suggest BMD improvements through phosphate binding. In the cross-sectional study (n = 321; 41% with osteoporosis), the only pharmacologic intervention associated with a reduced risk of osteoporosis was the use of CBPBs (OR 0.41 [95% CI, 0.21–0.81]; *p* = 0.011). The protective association was particularly evident in subgroups with dialysis vintage ≥10 years, serum albumin <35 g/L, concurrent active vitamin D analog therapy, and absence of proton pump inhibitor use [29]. In the 12 months cohort study (n = 65) only CBPBs were associated with significant BMD gains (Z scores lumbar spine 0.17 to 0.34, *p* = 0.002, hip −0.27 to −0.10, *p* = 0.006 [68].

**Kidney transplant:** Phosphate binders are seldom used in KTRs, as post-transplant mineral metabolism is typically dominated by hypophosphatemia driven by persistent hyperparathyroidism and elevated FGF23, particularly early after transplantation. In a retrospective cross-sectional study of 146 KTRs, fractures were common, and both lower serum phosphate levels and declining femoral neck T-scores were associated with increased fracture risk [69]. These findings highlight a potential role of hypophosphatemia in post-transplant bone fragility, warranting confirmation in prospective studies.

### 5.3. Safety

The safety profile of phosphate binders is generally very favorable. Calcium-based agents carry a risk of hypercalcemia and related complications, necessitating considered use. Observational data suggest a potential benefit of non-calcium-based binders with respect to cardiovascular outcomes and mortality; however, definitive evidence of superiority over calcium-based binders is lacking, as most trials are limited by short follow-up and low event rates (see also discussion of safety within the topic of calcium above). All binder classes are associated with gastrointestinal adverse effects. High pill burden and large tablet size remain important barriers to adherence. For lanthanum, existing safety data are generally reassuring, and long-term significance of tissue deposition remains under investigation. Prolonged use of aluminum-based binders is contraindicated owing to well-documented toxicity.

### 5.4. Pragmatic Clinical Approach

Hyperphosphatemia has repeatedly been shown to increase fracture risk in healthy kidney and CKD populations. In clinical practice, phosphate binders should be used to control progressive hyperphosphatemia, aiming toward values in the normal range. Evidence for fracture prevention is strongest in dialysis patients, where meta-analyses and large cohort studies support a protective effect. Data for non-dialysis CKD patients remain inconclusive; thus, focusing on the evolution of phosphate levels, other CKD-MBD parameters, severity, and duration of CKD and fracture risk is advisable. Use of calcium-based agents should be limited in patients with very high cardiovascular risk or positive calcium balance. Clinicians should account for gastrointestinal side effects, pill burden, and openly discuss benefits versus adherence challenges, tailoring binder choice and dosing to individual phosphate intake and overall cardiovascular and CKD-MBD profile. In kidney transplant recipients, routine use is generally not indicated given the predominance of post-transplant hypophosphatemia.

## 6. Calcimimetics

### 6.1. Rationale and Guidelines

Calcimimetics act as allosteric agonists of the calcium-sensing receptor on parathyroid cells, increasing sensitivity to extracellular calcium and thereby effectively lowering PTH secretion. Through PTH suppression and possibly also via direct inhibition of osteoclast activity, stimulation of osteoblast function, and upregulation of Wnt signaling, calcimimetics provide favorable effects on bone health, reducing high-turnover bone metabolism, and improving bone mineralization, density, and microarchitecture. Kidney guidelines recommend calcimimetics as part of the multimodal management of CKD-MBD for the control of PTH and its associated prognostic benefits, while no recommendation currently exists for specific bone health endpoints such as fractures or BMD [2,6].

### 6.2. Evidence on Fracture Prevention and Bone Mineral Density

**Advanced CKD:**

Secondary and tertiary hyperparathyroidism is an independent fracture risk factor in the non-dialysis CKD population. Participants from the US CRIC cohort (n = 3939) with a mean eGFR of 44 (SD 15, range 20–70) mL/min/1.73 m^2^ and follow-up times of 11.1 (4.8) years showed significantly increased fracture incidence if PTH was above the ULN (aHR 1.70 [95% CI, 1.10–2.65]; *p* < 0.05) [22]. The meta-analysis by Liu et al. found significantly elevated fracture risk with elevated FGF23 levels (eight studies, FGF > 58 pg/mL versus normal: RR 1.32 [95% CI, 1.06–1.66]; *p* = 0.015) [23].

Fractures: Evidence for the non-dialysis CKD population is very limited and without a suggestion of fracture risk reduction. Evans et al. provided Swedish cohort data (n = 3526) where in a mixed cohort of 435 patients (non-dialysis CKD n = 195, dialysis n = 190, KTRs n = 50) with a follow-up of 37 months (IQR 18, 61), use of cinacalcet was not associated with fracture risk (aOR 1.08 [95% CI, 0.59, 1.98]) [70].

BMD: A small placebo-controlled RCT that included four patients with stage 4 CKD and six patients on hemodialysis found cinacalcet to increase proximal femur BMD (0.945 to 0.961 g/cm^2^; *p* < 0.05) without an effect on lumbar spine; BMD change correlated with PTH reduction (R^2^ = 0.39; *p* < 0.05) [71].

**Dialysis:**

Secondary hyperparathyroidism is an independent fracture risk factor in the dialysis population. A meta-analysis of 11 studies found a significant increase in fracture risk in dialysis patients with PTH > 300 pg/mL compared to those with PTH levels of 150–300 pg/mL (RR 1.25 [95% CI, 1.20–1.31]; *p* < 0.001). Notably, PTH < 150 pg/mL was likewise associated with considerable increased fracture risk (10 studies: RR 1.41 [95% CI, 1.10–1.82]; *p* = 0.007) [23].

Fractures: The evidence comprises one very large RCT (EVOVE) [48], five meta-analyses (including seven, four, four, three, and two trials) [72,73,74,75,76], and a registry analysis [77]. Results are inconsistent; a reduction in fracture risk is likely.

In EVOLVE, the effect of cinacalcet vs. placebo on fracture incidence (secondary outcome) was investigated in 3883 dialysis patients with a median PTH of 694.5 (10th, 90th percentile: 362.0, 1707.0) pg/mL over a follow-up of up to 64 months. Results from unadjusted intention-to-treat analysis were not significant (relative hazard 0.89 [95% CI, 0.76–1.05]; *p* = 0.16). After adjustments for several imbalances and consideration of actual drug exposure, the relative hazard for fracture was 0.72 (95% CI, 0.58 to 0.90); *p* = 0.003. The effect appeared more pronounced in older patients [48]. Looking at the five meta-analyses, the largest and most recent investigation (seven trials, 6481 participants) found a large reduction in fracture incidence with calcimimetics compared to placebo or conventional treatment (RR 0.50 [95% CI, 0.29–0.88]; *p* = 0.02) with a number needed to treat of 47 [72]. However, some of the included studies have important limitations (low event rates, small sample sizes, varying concomitant therapies, short follow-up periods, and fracture as safety outcome). The other meta-analyses showed mixed results. Fracture risk reductions were reported by Cunningham et al. (four trials, n = 1184, RR 0.46 [95% CI, 0.22–0.95]; *p* = 0.04) [73] and Block et al. (three trials, fracture rate 1.6% vs. 2.9%) [75] but not by Palmer et al. (four trials, n = 4674, inclusion of non-dialysis patients possible) [74] and Wang (two trials, n = 3939) [76]. A propensity-score matched analysis of Japanese registry data (n = 7734) and found no differences in fracture incidence within 6 years, comparing parathyroidectomy versus cinacalcet (HR 1.14 [95% CI, 0.65–2.00]) [77].

BMD: The available evidence is limited to a small number of prospective studies (two controlled [78,79] and two uncontrolled studies [80,81]) with low sample sizes. In three [79,80,81] of the four studies, calcimimetics improved BMD, while the only negative trials had a short follow-up of 12 weeks [78]. Hung et al. (n = 60) and Khairallah et al. (n = 22) reported BMD gains after 6 months of cinacalcet (20.7%) and 9 months of etelcalcetide (3% at the spine and hip, and 7% at the femoral neck), respectively [80,81]. In 65 peritoneal dialysis (PD) patients, parathyroidectomy showed greater BMD increase compared to cinacalcet. However, cinacalcet was associated with significant 12 months lumbar spine T-score differences (0.30 [−0.13 to 0.63]; *p* = 0.04) and increased spine trabecular bone score (+10 ± 2%; *p* < 0.001) [79].

Histology: Two studies provided histological data. In Khairallah et al., bone biopsy in five patients showed a reduced bone formation rate (mean difference −25 ± 4 µm^3^/µm^2^ per year; *p* < 0.01) [81]. In Behets et al., among 77 patients with repeat biopsies after 6–12 months of cinacalcet, bone formation rate/tissue decreased (728 to 336 µm^2^/mm^2^/day), osteoblast perimeter/osteoid perimeter declined (17.4 to 13.9%), and eroded perimeter/bone perimeter decreased (12.7 to 8.3%). The number of patients with normal histology rose from none at BL to 20 at 12 months, while two developed adynamic bone with PTH < 150 pg/mL [82].

**Kidney transplant:**

In KTRs, calcimimetics are mainly used to manage persistent PTH autonomy with overt or persisting hypercalcemia.

Fractures: Evidence on fractures is scarce. In a small RCT, Evenepoel et al. reported 1/57 vs. 2/57 events (RR 0.50 [95% CI, 0.05–5.36]) [83]. Comparing parathyroidectomy with cinacalcet in 24 KTRs over a 5-years’ follow-up, Moreno et al. did not find significant differences in fragility fractures [84].

BMD: We are not aware of studies investigating calcimimetics and BMD in KTRs.

### 6.3. Safety

The safety of calcimimetics was comprehensively studied in a meta-analysis by Wang et al., including 25 studies and 23 RCTs (2 in CKD stages 3–4, 19 in dialysis, 1 in KTRs). Cinacalcet dosages ranged from 30 to 180 mg/day in 13 trials, and placebo served as control in 16 trials. Across 15 trials (n = 7685), cinacalcet modestly increased the risk of any adverse event (AE) compared with placebo or no treatment (RR 1.04 [95% CI, 1.00–1.09; *p* = 0.03; I^2^ = 68%). Specific AEs included hypocalcemia (18 trials, n = 7785: RR 8.48 [95% CI, 6.37–11.29; *p* < 0.001; I^2^ = 0%), nausea (17 trials, n = 7512: RR 2.12 [95% CI, 1.62–2.77; *p* < 0.001; I^2^ = 59%), vomiting (13 trials, n = 7331: RR 2.00 [95% CI, 1.79–2.24]; *p* < 0.001; I^2^ = 0%), diarrhea (11 trials, n = 6116: RR 1.17 [95% CI, 1.05–1.32]; *p* = 0.006; I^2^ = 0%), and muscle cramps or spasms (5 trials, n = 1692: RR 1.56 [95% CI, 1.08–2.25]; *p* = 0.02; I^2^ = 22%). Risk of hypotension was reduced (4 trials, n = 1611: RR 0.60 [95% CI, 0.42–0.84]; *p* = 0.004; I^2^ = 0%) [76].

### 6.4. Pragmatic Clinical Approach

Calcimimetics are a useful option for PTH control in secondary hyperparathyroidism and parathyroid autonomy across the CKD spectrum. Hyperparathyroidism is associated with increased fracture risk. In non-dialysis CKD patients, available data on fracture and BMD is insufficient to draw conclusions. In dialysis patients, evidence suggests a significant fracture risk reduction and BMD benefits, though data are inconsistent. In KTRs, use is mainly pragmatic for persistent PTH autonomy with hypercalcemia, as evidence for bone endpoints is lacking. Clinicians should monitor for hypocalcemia and gastrointestinal side effects, which are frequent. Overcorrection needs to be avoided as with hypoparathyroidism or ABD, fracture risk will increase again.

## 7. Bisphosphonates

**Rationale and Guidelines:** Bisphosphonates are an established and effective therapy for reducing fracture risk in osteoporosis. In advanced CKD, however, bone fragility arises through fundamentally different pathophysiological mechanisms, and evidence for bisphosphonate efficacy remains sparse. It is therefore unclear whether observed increases in BMD translate into true fracture risk reduction, or whether they merely reflect an expansion of bone mass within a structurally compromised skeleton. Concerns also include the potential for harm; suppression of turnover may predispose to ABD, a state associated with increased fracture and CV risk, and drug accumulation with declining renal function raises the possibility of exaggerated toxicity. Bisphosphonates are generally contraindicated below eGFR thresholds of 30–35 mL/min/1.73 m^2^. KDIGO recommends applying treatment strategies as in the general population in patients with CKD up to stage 3b and normal PTH levels. For more advanced stages, with obvious stigmata of CKD-MBD, or in post-transplant settings, consideration of bone biopsy is suggested before initiating bisphosphonates [2]. KDOQI underscores the need for bone histomorphometry before considering bisphosphonate therapy and reflects on integrating biopsy procedures into nephrology training programs [6]. The recommendation regarding a potential use of bisphosphonates has broadened from CKD 3b down to CKD 5D, integrating careful consideration of specific side effects and a weighing of how much diagnostic uncertainty can be accepted in this context [2]. This highlights the persisting uncertainty regarding risk–benefit balance and the need for better evidence to guide clinical decision-making in advanced CKD.

### Evidence on Fracture Prevention and Bone Mineral Density

**Advanced CKD:**

*Fractures:* A pooled analysis of nine clinical trials [85] (Miller et al., nine placebo-controlled trials, n = 8996), and a post hoc analysis of a large multicenter RCT [86] (Jamal et al., n = 6458), demonstrated a significant reduction in fracture risk with bisphosphonates versus placebo in patients with CKD. Treatment with risedronate and alendronate over mean follow-ups of 2 and 3 years resulted in vertebral fracture risk reductions of around 25–45% [85], as well as clinical and vertebral fracture risk reductions of OR 0.8 (95% CI, 0.70–0.9) and OR 0.54 (95% CI, 0.37–0.78), respectively [86]. Results of a meta-analysis by Whitlock et al. (seven placebo-controlled trials, n = 7428, follow-up 12 months) was marginally non-significant with a HR of overall fracture risk reduction with ibandronate vs. placebo of 0.78 (95% CI, 0.746–1.018) [87]. While these trial data appear reassuring, available evidence only marginally addresses the longstanding and challenging question of whether patients with advanced CKD also benefit. Miller et al. found fracture risk reductions independent of CKD stage (eGFR C & G < 80 vs. <50 vs. <30 mL/min.), with graphical reductions of −25%, −30%, and −45%, respectively. However, in the subgroup of CKD stage <30 mL/min. (n = 572), the median eGFR was not lower than 26.4 mL/min^.^ (IQR 23.1–28.5; range 13.2–29.9), and patients with hyperparathyroidism were excluded [85]. Jamal et al. also reported benefits independent of CKD stage; however, the comparison was dichotomized at eGFR > 45 vs. <45 mL/min/1.73 m^2^, and in the <45 mL/min/1.73 m^2^ subgroup (n = 581), no participants had an eGFR < 25 mL/min/1.73 m^2^ and very few had an eGFR < 30 mL/min/1.73 m^2^. Moreover, patients with PTH > 85 pg/mL or PTH > 65 pg/mL combined with abnormal calcium, alkaline phosphatase, or phosphate levels were excluded [86]. Whitlock et al. included merely 0.2% of participants (n = 15) with eGFR < 30 mL/min/1.73 m^2^, and among those with eGFR 30–45 mL/min/1.73 m^2^ (CKD G3b; 3%, n = 223), fracture rates were paradoxically increased with bisphosphonates (HR 3.86 [95% CI, 1.16–12.90]) [87].

In conclusion, for patients with CKD stage 5 (eGFR < 15 mL/min/1.73 m^2^), no data are available. For patients with CKD stage 4 (eGFR of 15–30 mL/min/1.73 m^2^), only the pooled analysis by Miller et al. provides data from a sufficiently large number of participants showing significant fracture risk reductions (−45%) in 572 patients with eGFR of 13–29 mL/min/1.73 m^2^ (around 150 patients with eGFR 15–25 mL/min/1.73 m^2^) [85]. No data are available for patients in advanced CKD stages exhibiting characteristic features of clinically significant CKD-MBD.

*BMD:* The aforementioned studies likewise demonstrate significant BMD gains, albeit harboring the same limitations of very low CKD stage 4 representation and the absence of individuals with CKD-MBD. Miller et al. showed significant mean BMD improvements across all three CKD stages (e.g., for the severe group [eGFR C & G < 30 mL/min] vs. placebo: 4.23% [1.82] vs. −1.37% [1.72]; *p* < 0.001) [85] and Jamal et al. observed significant thoracic spine BMD increases in patients with eGFR < 45 mL/min/1.73 m^2^ (5.6% [95% CI, 4.8–6.5]) [74]. Whitlock et al. reported significant lumbar spine changes (n = 2617, β = 0.0245 [SE 0.00253]; *p* < 0.0001) [87]. Three additional studies on alendronate merit mention: one RCT (n = 51; mean eGFR 33.8 mL/min/1.73 m^2^) reported lumbar spine T-score difference of 0.3 (95% CI, 0.03–0.6; *p* = 0.04) vs. placebo at 18 months follow-up [88]; one propensity score-matched retrospective cohort (n = 213; mean eGFR 37.1 ± 9.1 mL/min/1.73 m^2^) showed annualized BMD gains vs. placebo ranging from 2.12% (95% CI, 0.98–3.25) at the spine to 2.65% (95% CI, 1.32–3.99) at the femoral neck [89]; and one prospective cohort (n = 50) exhibited small but significant T-score improvements (−3.17 [0.24] to −3.16 [0.25]; *p* < 0.05) over six months in the CKD stage 4 group (mean eGFR 23.5 ± 3.64 mL/min/1.73 m^2^) [90].

**Dialysis:**

*Fractures:* We are not aware of studies investigating the effect of bisphosphonates on fracture incidence in dialysis patients.

*BMD:* Two very small prospective cohort studies investigated the effect of 12-months i.v. bisphosphonates on BMD [91,92]. In 16 patients with osteopenia, ibandronate 2 mg monthly was associated with significant mean T-score improvements of 0.3 points [91]. In 13 patients with secondary hyperparathyroidism, pamidronate 60 mg every 2 months was associated with mean T-scores increases of 33%. Notably, this significant BMD response was observed in a high-turnover osteodystrophy setting with PTH > 500 ng/mL being a central inclusion criterium [92]. In both studies, PTH and electrolytes did not show concerning variations.

**Kidney transplant:**

*Fractures:* Solid evidence indicates no reduction in fracture risk with bisphosphonates during the first 6–48 months after transplantation. Across three meta-analyses (23 RCTs) [93,94,95], two systematic reviews (18 RCTs and quasi-RCTs) [96,97], and two retrospective cohorts [98,99], no signal of significant fracture reduction was observed. Given the typical follow-up of 12–24 months (range 3–48 months) and the dedicated strain on osteometabolic systems during the early post-transplant phase, these findings cannot be extrapolated to later periods. It is conceivable that, depending on cumulative uremic exposure and CKD-MBD phenotype, long-term KTRs may partly resemble non-KT CKD patients with potentially better response to bisphosphonate therapy; however, data are lacking.

*BMD:* Credible evidence derives from three meta-analyses (34 RCTs, 1855 patients) [93,95,100], two systematic reviews (20 RCTs, 847 patients) [96,97], and two retrospective cohorts (436 patients) [99,101]. Clinical trial results are inconsistent. The most recent meta-analysis found no BMD improvement over 12 months (10 RCTs, 592 patients) [93], whereas the two other meta-analyses reported clinically meaningful BMD increases of mean 6–7.4% (12 RCTs, 621 patients follow-up 3.6–13.2 months) [95] and 7.24% (95% CI, 3.73–10.69), respectively (4 RCTs, 152 patients) [100]. A 2019 review (13 RCTs, 579 patients) found no effect on BMD with bisphosphonates versus placebo [96], while a 2005 review (7 RCTs, 268 patients) found meaningful BMD gains of around 7% [97]. A potential long-term benefit is suggested by a recent 4-year retrospective cohort (n = 254), showing clinically relevant mean T-scores improvements of 0.63 points with bisphosphonates vs. controls [101].

**Safety of bisphosphonates**

*Malignant Disease—i.v. Bisphosphonates:* Most evidence on bisphosphonate-associated nephrotoxicity originates from patients without relevant pre-existing CKD that suffered from malignant bone-metastatic disease (often multiple myeloma, also prostate, breast, or other solid cancers), receiving high-dose intravenous zoledronate or pamidronate. Data derive primarily from two pharmacovigilance databases (US and France) [102,103], supplemented by one phase III RCT [104], one retrospective cohort study [105], and ten biopsy-based case reports [106]. Standard regimens (if specified) included zoledronate 4–8 mg every 3–4 weeks or pamidronate 90–360 mg monthly. However, pooled data from five RCTs (n = 2858) in malignant bone disease (multiple myeloma or bone metastases) found no consistent nephrotoxicity signal for pamidronate, zoledronate, or ibandronate at standard or intensified doses, with up to 2 years’ follow-up [107,108,109,110,111].

The US FDA database reported 72 cases of severe renal failure with zoledronate over 18 months, with mean creatinine increasing from 150 to 575 μmol/L within ~56 days after treatment initiation (mean 2.4 infusions) [102]. The three-years’ French registry data recorded seven cases of AKI within 1–120 days after bisphosphonate exposure [103]. Nephrotoxicity risk correlated with dose and infusion duration. In the phase III RCT, sequential protocol modifications (8 mg over 5 min → 8 mg over 5–15 min → 4 mg over 5–15 min) reduced incidence of renal damage to match pamidronate [109]. In a cohort of 122 prostate cancer patients, 23.8% developed renal impairment after ≥1 dose of zoledronate [105]. In 22 biopsy-proven cases, presentations included AKI (n = 18), nephrotic syndrome (n = 18), and nephrotic-range proteinuria (n = 3), with histology showing collapsing FSGS (n = 15), FSGS-NOS (n = 4), and MCD (n = 4) [106]. The above-mentioned evidence found advanced cancer, prior bisphosphonate exposure, NSAIDs, age, pre-existing kidney disease, hypertension, and smoking as risk factors for kidney toxicity upon bisphosphonate use. Recovery was often incomplete.

Malignant disease with pre-existing CKD: Evidence here is very scarce. Two small studies (total n = 28) in multiple myeloma patients found no renal decline with i.v. ibandronate 6 mg over 30 min every 3–4 weeks.

*Osteoporosis—Healthy Kidneys:* Looking at four large RCTs (in total, 10,976 patients) [112,113,114,115] investigating oral bisphosphonates (alendronate, ibandronate, or risedronate), and five RCTs (in total, 11,670 patients) [116,117,118,119,120] investigating various regimes of i.v. bisphosphonates (ibandronate or zoledronate), no increases in kidney adverse events have been found within follow-up times of 1–3 years. There is one recent case report documenting collapsing FSGS with AKI and nephrotic syndrome after oral alendronate 70 mg weekly [121].

*Osteoporosis—Chronic Kidney Disease—Oral Bisphosphonates:* For the indication of osteoporosis, nephrotoxicity data mainly originates from patients with CKD receiving oral bisphosphonates. Similar to the evidence in malignancy outlined above, nephrotoxicity reports stem from two database analyses [122,123] and single case reports [124,125,126,127] whereas clinical trial evidence does not report kidney toxicity [85,86,87,88,128].

The two database studies applied propensity-score matching analyses (CPRD GOLD UK: n = 2447 vs. 8931, SIDIAP Catalonia: n = 1399 vs. 6547, and CPRD GOLD: n > 20,000 vs. >200,000 patients) and found a 12–14% increased risk of CKD progression over 3–4 years in oral bisphosphonate users vs. non-users. The subdistribution HRs (95% CIs) were 1.14 (1.04–1.26), 1.15 (1.04–1.27), and 1.12 (1.02–1.24), respectively. The incidence of AKI was not increased [122,123]. Four case reports [124,125,126,127] (oral alendronate 35–70 mg weekly) describe FSGS (n = 4, 2× collapsing, 2× NOS), AKI (n = 2), severely progressive CKD (n = 1), and proteinuria (n = 1); recovery was complete (n = 2), partial (n = 1), or absent (n = 1), respectively. Looking at the trial evidence from two meta-analyses (seven trials, n = 7428 and nine trials, n = 8996) [124,125,126,127], two RCTs (post hoc n = 6458 and n = 51) [86,88], and one prospective cohort (n = 50) [90], no kidney toxicity was detected analyzing the effect of oral alendronate, ibandronate, and risedronate with follow-up times of 1, 3, 3, 1.5, and 0.5 years, respectively.

*Osteoporosis—Chronic Kidney Disease—i.v. Bisphosphonates:* Virtually no data exist. We found a report of two cases (eGFR 66 and 44 mL/min/1.73 m^2^), where AKI with ESKD developed after 5 mg of i.v. zoledronate administered over 15 and 30 min, respectively. These two patients recovered partly but remained with advanced CKD (eGFR 38–45 and 15–20 mL/min/1.73 m^2^) [129].

*Dialysis:* For the dialysis population, we are only aware of two very small prospective cohort studies (n = 16 and n = 13) in patients with osteopenia (PTH > 2× ULN) and patients with secondary hyperparathyroidism (PTH > 500 pg/mL). With i.v. ibandronate 2 mg monthly over 3 months and i.v. pamidronate 60 mg every 2 months over 12 months, respectively, no kidney adverse events were found apart from mild calcium reductions [91,92].

We are not aware of studies investigating p.o. bisphosphonates in dialysis patients.

*Kidney Transplant Recipients:* A meta-analysis of 12 placebo-controlled RCTs (n = 621) found no effect of p.o. or i.v. bisphosphonates (clodronate, pamidronate, alendronate, ibandronate, risedronate, and zoledronate) on allograft function [95]. A systematic review (45 trials, n = 2698) likewise reported no kidney safety concerns [96]. Intravenous or p.o. bisphosphonates were initiated within 3 weeks post-transplant regardless of BMD and median follow-up was 12 months. In a retrospective cohort, prolonged oral bisphosphonate use (alendronate, ibandronate, or risedronate for 3.9 ± 2.3 years, starting 1.1 ± 2.4 years after transplantation), showed no adverse graft effects in users (n = 35) vs. non-users (n = 219) [101]. Interestingly, a prior systematic review found reduced rejection risk (RR 0.59, 95% CI, 0.39–0.90) in the bisphosphonate group, possibly reflecting immunomodulatory and anti-inflammatory properties [97].

Bisphosphonates and Adynamic Bone Disease: It is well established that bisphosphonates reduce bone turnover via inhibition of osteoclast activity, thereby increasing bone mass and effectively preventing fractures in individuals with preserved kidney function. However, prolonged or excessive suppression of bone turnover may ultimately compromise skeletal integrity. ABD has been unequivocally associated with increased fracture risk and cardiovascular morbidity. Given that the incidence of ABD is already high in patients with CKD, concerns are justified that bisphosphonate therapy—with its inherently long skeletal half-life and risk of accumulation in renal insufficiency—may expose this population to unnecessary harm. The magnitude of this risk, however, remains uncertain.

A biopsy study in 13 CKD patients (including two KTRs; mean eGFR 46 mL/min/1.73 m^2^, mean iPTH 57 pg/mL) who had received bisphosphonates for 4 to >60 months found ABD in all participants. While very concerning, these findings must be interpreted with caution due to the study’s cross-sectional design, lack of baseline biopsies or laboratory data, and absence of a control group [130].

A prospective trial of 16 dialysis patients with PTH > 2× ULN treated with intravenous ibandronate (2 mg every 4 weeks for 48 weeks) reported an initial rise in PTH followed by a decrease to 18.99 pmol/L at week 48, non-significant to BL. Bone turnover markers declined (unspecified), and calcium remained stable with calcitriol doses only modestly increased (1.5 to 1.83 µg/week) [91]. Another prospective study in 13 dialysis patients with iPTH > 500 pg/mL and moderate hypercalcemia tested pamidronate 60 mg every 2 months for one year. PTH, which increased in all patients at three months, only dropped after increasing calcitriol dose (in 10 patients PTH decreased by >50%; 3 patients had no response) [92].

In 59 KTRs, a biopsy-based RCT found 50% of the participants exhibiting ABD already at BL. After six months of pamidronate combined with vitamin D and calcium supplementation, 100% of treated patients had developed ABD. From the controls (treated with vitamin D and calcium) 50% continued to have or developed decreased bone turnover [131].

Taken together, these studies illustrate both the potential for bisphosphonates to exacerbate low-turnover states and the challenges in interpretation due to small sample sizes, lack of long-term outcome data, and pre-existing risk of ABD in CKD and KTRs. Beyond bisphosphonate exposure, additional risk factors for ABD (see above) must be carefully considered in individual therapeutic decision-making.

**Pragmatic Clinical Approach:**

For non-dialysis CKD patients with eGFR < 15 mL/min/1.73 m^2^ or for those with significant features of CKD-MBD (elevated PTH and phosphate, reduced calcium, etc.) no data on fracture risk exist. In these patients, it is unknown if bisphosphonates are for good or might harm and it is uncertain whether extrapolation from data of higher CKD stages is permissible. For patients with eGFR 15–30 mL/min/1.73 m^2^ without CKD-MBD stigmata, treatment with bisphosphonates in high fracture risk situations can be justified, as limited clinical trial data suggest a significant improvement of fracture incidence and BMD.

For dialysis patients, no data on bisphosphonates and fracture risk exist. Two very small studies found significant improvements of BMD with i.v. bisphosphonates. It is unclear if bisphosphonates will be of benefit or harm here. If bisphosphonates are desired for fracture prevention in dialysis patients, evidence from non-dialysis CKD populations suggests that risk/benefit balance might be more beneficial in those with shorter duration in advanced CKD stages (less CKD-induced, multidimensional bone destruction) and those without risk factors for or a course suggesting ABD.

For KTRs, solid clinical trial evidence indicates no reduction in fracture risk with bisphosphonates during the first 6–48 months after transplantation. The evidence regarding BMD is conflicting with convincing data for and against this surrogate endpoint efficacy of bisphosphonates in KTRs. KTRs constitute a distinct population with highly unique risks for bone health. However, in the long-term, aspects of bone health may partly resemble non-KTRs CKD patients with, by analogy, a potentially better response to bisphosphonates beyond a certain time after transplantation; however, data are very scarce.

Independent of CKD stage, in a clinical context where long-standing advanced kidney disease and corresponding biochemical hallmarks strongly suggests advanced renal osteodystrophy, it remains unclear whether an increase in BMD translates into improved skeletal stability, or whether it merely reflects a densification of an already multidimensionally unstable framework, further increasing fragility by suppressing residual turnover.

In the majority of cases, bone biopsy remains the diagnostic solution.

The overall risk of bisphosphonate-associated renal toxicity is small and comprises chronic decline, acute kidney injury, and nephrotic syndrome (FSGS and MCD). Risks are particularly pronounced in patients with malignant disease receiving high cumulative doses of intravenous bisphosphonates. In CKD patients with osteoporosis, two large database studies report a 12–14% risk of chronic progression over 3–4 years, whereas clinical trial data found no renal adverse events. Virtually no safety data exist for dialysis patients. In KTRs, robust evidence shows no effect on graft function (p.o. or i.v.). Thus, the risk for renal toxicity is small overall but not negligible. Oral risedronate at a reduced dose (35 mg every two weeks), with discontinuation in the event of renal deterioration, is a potential option. Whether this strategy reduces renal toxicity or whether drug withdrawal upon functional decline prevents further progression, however, remains unproven. The risk of developing ABD under bisphosphonate therapy—and thereby even increasing the risk of fractures and CV events—is mechanistically plausible and supported by limited evidence. The magnitude of this risk, however, remains unclear. If a risk of ABD is already apparent (Table 2), bisphosphonates are not advisable. Crucially, individual pre-existing risk factors for ABD need to be considered. In case of uncertainty, perform bone biopsy.

## 8. Denosumab

### 8.1. Rationale and Guidelines

Denosumab is a fully human monoclonal IgG2 antibody that effectively binds to the receptor activator of nuclear factor κB ligand (RANKL), a pivotal regulator of osteoclast formation, activity, and survival. By its inhibition, denosumab effectively suppresses bone resorption resulting in increased bone mass and strength in both cortical and trabecular compartments. Unlike bisphosphonates, its elimination is independent of renal function; consequently, no dose adjustment is required in CKD or dialysis patients, and there is no risk of drug accumulation and immunoglobulins are not removed by dialysis (IgG ≈ 150 kDa). Denosumab represents a valuable therapeutic option for patients with osteoporosis at high fracture risk, particularly when bisphosphonates are contraindicated, poorly tolerated, or ineffective. However, given its antiresorptive mechanism, denosumab may, similar to bisphosphonates, reduce bone turnover and thereby potentially increase the risk in CKD patients of inducing or aggravating low bone turnover with the risk of ABD. The true magnitude of this risk and the balance between potential fracture prevention and turnover suppression remain uncertain. Importantly, as with bisphosphonates, an increase in bone density achieved with denosumab in CKD may not necessarily translate into greater mechanical competence, since the multiple CKD-MBD-specific disturbances of bone quality are not addressed. Current guidelines [2,19] acknowledge denosumab as a potential therapeutic option in CKD patients, but emphasize the absence of robust fracture outcome data and the associated risk of hypocalcemia.

### 8.2. Evidence on Fracture Prevention and Bone Mineral Density

**Advanced CKD:**

Limited positive evidence in non-severe CKD stages comes from one post hoc analysis of a large phase III RCT (Fractures, BMD) [132] and two prospective cohorts (BMD) [133,134].

*Fractures:* A post hoc analysis of the large, 3-years’ phase III FREEDOM trial in postmenopausal osteoporosis found considerable vertebral fracture reductions with denosumab compared to placebo in CKD stage 1 (n = 842), stage 2 (n = 4069), and stage 3 (n = 2817) with ORs (95% CI) of 0.33 (0.16–0.66), 0.23 (0.15–0.34), and 0.38 (0.26–0.59), respectively. The CKD stage 4 group was small (n = 73) and did not show reduced fracture incidence with denosumab versus placebo (OR 0.31 [95% CI, 0.02–5.08]). No participant with CKD stage 5 was included. Non-vertebral fractures were only reduced in the CKD stage 2 group [132].

*BMD:* The post hoc analysis of the FREEDOM trial showed significant and clinically meaningful BMD increases with denosumab compared to placebo at lumbar spine, femoral neck, and total hip across all CKD stages with effect sizes between 5.1% and 9.0%. Only in CKD stage 4 and only at the lumbar spine locus, the BMD gains were just not significant (5.0% [95% CI, −0.8 to 10.8]) which might be due to sample size [132]. In a prospective study of 203 CKD patients, Kunizawa et al. found mean annual BMD increases at lumbar spine and femoral neck of 7.5% (10.2); *p* < 0.01 and 3.1% (9.5%); *p* < 0.05 (radius not significant). Small numbers of patients with advanced CKD were included (CKD stage 3b: n = 35, CKD stage 4 + 5: n = 20), but without stigmata of advanced CKD-MBD (mean PTH 54 [IQR 39–74] pg/mL, mean BAP 14.4 [SD 6.4] μg/L) [133]. A small but controlled one-year prospective cohort study (n = 27), including participants with a mean eGFR of 30.1 (12.3) mL/min/1.73 m^2^ and a mean PTH of 107 (71.4) pg/mL, showed significant BMD differences compared to controls of 3.6 (3.2) vs. −0.7 (4.4) %; *p* = 0.033 at femoral neck and 3.4 (3.8) vs. −1.9 (2.1) %; *p* = 0.001 at total hip. BMD at lumbar spine was not significant [134].

**Dialysis:**

Limited evidence comes from one large observational study (factures) [135], a small meta-analysis of observational data (BMD) [136], and two prospective [133,137] and one retrospective [138] study (BMD).

*Fractures:* In a recent 3-years’ observational study employing a trial emulation approach, denosumab (n = 658) compared to bisphosphonates (n = 374), lowered the fracture risk (composite all anatomical sites) by 45% (weighted RR 0.55 [95% CI, 0.28 to 0.93]) with a weighted risk difference of −5.3% (95% CI, −11.3% to −0.6%). Inclusion comprised 37% males and 18% of participants had a history of a fracture. Severity of CKD-MBD was not specified, and dialysis vintage was 1.5 years. Approximately 4% of participants received peritoneal dialysis; however, restricting the analysis to HD patients did not alter results. The authors acknowledged that the estimates lacked precision and warranted confirmation in future studies [135].

*BMD:* The observational evidence consistently provides significant and clinically relevant improvements of BMD with denosumab. A small meta-analysis of six observational studies (84 participants) with follow-up times of 4–24 months found T-score improvements of 0.39 (95% CI, 0.10–0.69) at the lumbar spine and 0.79 (95% CI, 0.60–0.98) at the femoral neck [136]. In a prospective Japanese cohort of 83 patients receiving denosumab, annual BMD improvements of 6.7% (11.1), *p* < 0.01 at the lumbar spine and of 4.3% (7.9), *p* < 0.01 at the femoral neck were observed. At baseline, iPTH was 132 pg/mL (IQR 56–217), bone-specific AP was 19.6 μg/L (12.4), and TRACP-5b was 496 mU/dL (299–797) [133]. A small prospective, controlled cohort study in male HD patients (n = 37) reported a mean 12-month BMD change of +2.6% (4.4) with denosumab compared to −4.5% (7.7) without denosumab (*p* < 0.001) [137]. A 5-years’ retrospective analysis (n = 124) investigated DEXA-derived cortical and trabecular 3D compartments at the hip region, before, during, and after denosumab. Areal, cortical (volumetric, surface, and thickness) and trabecular BMD increased with median changes between 3.2% and 9.8% with a trend of substantial decline after discontinuing the therapy [138].

### 8.3. Kidney Transplant:

*Fractures:* For the KTR population, we are not aware of studies investigating the effect of denosumab on fracture outcomes.

*BMD:* Very limited existing evidence shows significant BMD improvements with denosumab in KTRs with good allograft function. In a small meta-analysis (one trial, four cohort studies, n = 162, majority with BL eGFR of ≥30 mL/min/1.73 m^2^), standardized mean BMD (g/cm^2^) differences after 6–12 months of denosumab were 3.26 (95% CI, 0.88–5.64); I^2^ = 94% and 1.83 (95% CI, 0.43–3.22); and I^2^ = 54% at the lumbar spine and the femoral neck, respectively. Corresponding T-score changes were 0.92 (95% CI, 0.58–1.25) and 1.14 (95% CI, 0.17–2.10) [139]. A prospective controlled cohort investigated long-term effects in 46 KTRs (males and females) with osteoporosis (median FRAX 10-year fracture risk 16% [10.4, 21.1]), good allograft function (creatinine 115.8 [39.8] µmol/L), and controlled CKD-MBD parameters (PTH 123.5 [96.0] pg/mL, corrected calcium 2.4 [0.125] mmol/L, alkaline phosphatase 83.4 U/L [43.5]). Sixty-one percent had a history of dialysis and median time form transplant to start denosumab was 4 years (range 0–24 months). From BL to 4 years follow-up, BMD decreased in controls (LS −3.0% [7], *p* = 0.041; TH −6.3% [9.2]; *p* = 0.003; FN −6.7% [9.3], *p* = 0.003) but increased in the denosumab group (LS 9.0% [10.7], *p* < 0.001; TH 3.8% [7.9]; *p* = 0.041) with significant and clinically meaningful between-group differences at all sites [140].

### 8.4. Safety of Denosumab

*Non-CKD:* For patients with healthy kidneys, the large FREEDOM trial [141], its 10 years extension [142], and meta-analyses of RCTs [143,144,145] showed an overall favorable safety profile of denosumab. The FREEDOM trial (n = 7868) investigated denosumab versus placebo and found no increase in the risk of cancer, infection, cardiovascular disease, delayed fracture healing, hypocalcemia, or osteonecrosis of the jaw [141] with incidence for adverse events remaining stable throughout the 10 years’ trial extension [142]. A recent meta-analysis of 11 RCTs including 5545 participants compared the safety profile of denosumab against bisphosphonates over a 12–24 months treatment period. While they found less withdrawals due to AEs (RR 0.49 [95% CI, 0.34–0.71]), the risk of CV events was increased (5P MACE: RR 2.05 [95% CI, 1.03–4.09], CV AEs: RR 1.61 [95% CI, 1.07–2.41]) as well as the risk of infections (all infections: RR 1.14 [95% CI, 1.02–1.27], respiratory tract infections: RR 1.56 [95% CI, 1.08–2.25]) [143]. Diker-Kohen et al. investigated the risk of infection including 33 trials (22,253 participants) comparing denosumab to any control treatment. Whereas the overall risk for any infection or related mortality was similar to comparator groups, the incidence of infection-related severe AEs (SAEs) was increased (RR 1.21 [95% CI, 1.04–1.40]; I^2^ = 0%) with a significant increase in ear, nose, and throat (RR 2.66 [95% CI, 1.20–5.91]), as well as gastrointestinal infections (RR 1.43 [95% CI, 1.02–2.01]) [145].

*Advanced CKD:* For the population of patients with advanced CKD, the safety profile of denosumab is not well studied. Post hoc analyses of the FREEDOM trial and its 10 years’ extension did not show major differences in the incidence of AE (CKD stage 4: 35 [97.2%] vs. 35 [94.6%]) or SAE (CKD stage 4: 15 [41.7%] vs. 13 [35.1%]), including CV events and hypocalcemia between denosumab and placebo stratified by kidney function. However, numbers of participants with advanced CKD were low (FREEDOM: CKD stage 4: n = 73, CKD stage 5: n = 0, FREEDOM extension: CKD stage 3b: n = 66, CKD stage 4: n = 9 [not included]) [141,142]. In contrast, real-world settings document a relevant risk of hypocalcemia and the FDA issued a black box warning in January 2024 for severe hypocalcemia in patients with advanced CKD treated with denosumab. In a prospective cohort of 203 CKD patients, risk of hypocalcemia (2.0 to <2.12 mmol/L) was evident and clearly progressive across CKD stages (CKD stages 1–2: 1%, CKD stage 3a: 2.3%, CKD stage 3b: 8.6%, CKD stage 4–5: 25%). The prevalence in advanced CKD patients (CKD stage 4–5) was comparable to those on hemodialysis, which are known to harbor the highest risk (17%). Median (IQR) days until hypocalcemia were 6 (3–7). Hypocalcemia below 2.0 mmol/L did not occur in any CKD stage [133]. In a small, controlled cohort study (n = 27) where participants had a mean eGFR of 30.1 (12.3) mL/min./1.73 m^2^ and took calcium carbonate (1000–1250 mg per day) plus vitamin D (800–1000 IU per day), no hypocalcemia was found during the 1 year study period, underscoring the suggestion that the risk of hypocalcemia is associated with declining kidney function and that preventative measures work [134]. With regard to denosumab-related infections, it cannot be excluded that patients with CKD harbor an increased risk, especially if immunocompromised, including steroid doses >2.5 mg/day [146].

*Dialysis:* First and foremost, the risk of hypocalcemia needs to be addressed, which is especially pronounced in dialysis patients, who suffer from progressive difficulties in timely compensation of calcium imbalances. For denosumab compared to oral bisphosphonates, a large cohort study (n = 2804) showed weighted incidence rates of 41.1% vs. 2.0% for severe hypocalcemia (cCa < 1.88 mmol/L or hospitalization) and 10.9% vs. 0.4% for very severe hypocalcemia (cCa < 1.63 mmol/L or emergent care), translating to a RR of 20.7 (95% CI, 13.2–41.2) and 26.4 (95% CI, 9.7–449.5), respectively [147]. A small meta-analysis of six observational studies (n = 84) found a pooled estimated hypocalcemia incidence of 42% (95% CI, 29–55%, I^2^ = 0%) [136]. The risk of hypocalcemia in patients on hemodialysis is significantly greater compared to those of non-dialysis CKD stages. Kunizawa et al. found prevalence rates of hypocalcemia (<2.12 mmol/L) in hemodialysis (n = 121) versus non-dialysis CKD patients (n = 203) to be 35.6% versus 5.4% (*p* < 0.001). The prevalence of serum calcium between 1.75 and <2.0 mmol/L was 18% in hemodialysis patients, compared to 0% in the CKD group [133]. Hypocalcemia seems to develop somewhat later than in non-dialysis CKD patients (meta-analysis [136]: 7 to 20 days, nadir during first 2 weeks up to 2 months. Cohort [133]: 7 (5–21) days. Cohort [137]: Mean cCa BL to 7 days to 1 months: 2.30 [0.12] to 2.12 [0.12] to 2.30 [0.22] mmol/L). Hypocalcemia may trigger considerable fluctuations of PTH [148] with values up to several thousand pg/mL. Although very high iPTH values trigger discomfort amongst care teams, they largely seem to be an expression of compensatory mechanisms following heavily blocked bone metabolism, indicating pronounced needs for temporary calcium and/or vitamin D substitutions. Available data do not show an increased risk of CV events. However, a large observational study from Japan (n = 1032) showed borderline significant results for MACE compared to bisphosphonates (weighted 3-year RR 1.36 [95% CI, 0.99–1.87], weighted 3-year RD 8.2% [95% CI, −0.2% to 16.7%]) that need to be confirmed [135]. Only one small study evaluated the risk of infection and found respiratory tract infections to hold second place (4.17%) after hypocalcemia (33.33%) among the most common adverse events [149].

*Kidney Transplant:* In KTRs, the risk of hypocalcemia has also been documented. A meta-analysis comprising 162 KTRs found 12 episodes in 39 denosumab-treated patients versus 1 episode in 42 controls. Included cohort studies showed a pooled hypocalcemia incidence with denosumab of 1.7% (95% CI, 0.4–6.6%). All episodes were mild and asymptomatic, but the majority of patients required calcium and vitamin D supplements. It is important to mention that participants had good allograft function (majority with BL eGFR ≥ 30 mL/min/1.73 m^2^) [139]. A recent controlled cohort study of 46 postmenopausal KTRs with osteoporosis (mean creatinine 115.8 (39.8) µmol/L, iPTH 123.5 (96.0) pg/mL, median time form NTX to denosumab 4 years (range 0–24 months), h/o fragility fractures: n = 7/23, median FRAX 10 years fracture risk 16% [10.4, 21.1]) did not show significant differences regarding hypocalcemia, allograft function, and rejection rates during 4 years treatment duration [140].

Independent of the CKD population, the substantially increased rebound fracture risk after discontinuation of denosumab must always be taken into account. Before initiating denosumab, this consideration must be incorporated into the treatment decision, including the options of a prolonged duration of therapy or subsequent treatment with bisphosphonates.

### 8.5. Pragmatic Clinical Approach

For CKD patients, limited but positive evidence for fracture reduction and BMD improvements with denosumab is available, however, only for non-advanced CKD (stage 3). For dialysis patients, evidence suggests significant BMD gains. Evidence on fracture reduction is limited and uncertain; however, a significant effect is possible. For KTRs, no data regarding fracture risk is available. Limited evidence on significant BMD improvements seems promising. Genuine safety concerns primarily relate to hypocalcemia. The risk is clearly dependent on kidney function, rising substantially in CKD stages 4 and 5 and reaching its peak in dialysis patients. During the first two weeks and up to one to two months following denosumab administration, calcium nadir must be closely monitored and temporary calcium and vitamin D supplementation should be initiated preemptively. If recurrent infections occur, a causal association is possible and risk/benefit balance should be reconsidered. Analogous to bisphosphonates (see above), denosumab carries the risk of inducing ABD, with its attendant increase in risk of fractures and CV events. The magnitude of the risk for ABD development under denosumab, however, remains uncertain. The post-denosumab rise in PTH levels can be worryingly high. While this is generally regarded as a compensatory and necessary response, signaling the need for calcium and vitamin D supplementation rather than the need for suppressive intervention, the overall effect of repeated cycles is unknown. Upon discontinuation of denosumab, rapid BMD loss may occur (e.g., below baseline values), and this is associated with increased fracture risk, including multiple vertebral fractures. Even though long-term data in patients with CKD have become increasingly available, treatment duration and options for subsequent therapy to mitigate the rebound fracture risk after denosumab (e.g., bisphosphonates), should be considered prior to therapy initiation. In this context, the decision for bisphosphonate therapy is somewhat “easier”, as the primary objective is not only fracture risk reduction per se, but the prevention of the rebound phenomenon following discontinuation of denosumab.

## 9. Romosozumab

### 9.1. Rationale and Guidelines

Romosozumab is a humanized monoclonal IgG2 antibody that induces osteoanabolic changes via the inhibition of sclerostin. Romosozumab stimulates osteoblastic bone formation while suppressing osteoclastic resorption. It increases trabecular and cortical bone mass, improves bone microarchitecture, and enhances bone strength. Romosozumab represents a promising, powerful therapeutic option for severe postmenopausal osteoporosis. There is growing evidence and use within additional clinical contexts characterized by high fracture risks with romosozumab providing primary anabolic support or further benefit in therapy-refractory cases. To date, romosozumab has not been incorporated into nephrology guidelines.

### 9.2. Evidence on Fracture Prevention and Bone Mineral Density

**Advanced CKD:**

The available evidence is very limited and does not include participants with eGFR < 30 mL/min/1.73 m^2^. Nevertheless, for CKD 1–3, data are of high scientific value and demonstrate remarkable reduction in fracture incidence.

*Fractures:* A post hoc analysis of two double-blind, multicenter phase III randomized controlled trials investigated the 12-month effects of romosozumab versus placebo (FRAME; n = 7147) or versus alendronate (ARCH; n = 4077) in postmenopausal women. Treatment effects were similar across different levels of kidney function. Among participants with CKD stage 3, relative risk of new vertebral fractures was 72% (95% CI, 14–91; *p* = 0.017) compared to placebo and 51% (95% CI, 5–75; *p* = 0.04) compared to alendronate [150].

*BMD:* In the post hoc analysis cited above, least-squares mean changes from baseline to 12 months in participants with CKD stage 3 were 10.9% (95% CI, 10.4–11.4; *p* < 0.001) at the lumbar spine and 4.6% at the femoral neck compared to placebo. Relative to alendronate, changes were 8.1% (95% CI, 7.3–8.9; *p* < 0.001) at the lumbar spine and 2.7% at the femoral neck. Across all categories of kidney function, increases in bone mineral density were significantly greater with romosozumab than with control treatments [150].

For patients with advanced CKD, hence for non-dialysis patients with potentially significant CKD-MBD osteometabolic states, data are absent.

**Dialysis:**

Evidence comprises limited data from Japanese cohorts where romosozumab was not associated with fracture reductions but with increases in BMD.

*Fractures:* The main evidence has been provided by Sato et al. with a 12-months prospective controlled study of 131 patients with severe osteoporosis. A total of 20.8% had pre-existing fragility fractures, 61.5% had a history of bisphosphonate treatment, mean PTH was 152.3 pg/mL (172), and 37% were on calcimimetics. Incidence of fragility fractures was not significantly different between groups (romosozumab: 3/96 [3.8%], all prox. Femur. Controls: 3/55 [5.5%], thoracic spine, lumbar spine, and prox. femur). Baseline BMD at the femoral neck was lower in the romosozumab group (0.439 ± 0.073 vs. 0.512 ± 0.105 g/cm^2^; *p* < 0.0001) [151]. In a small prospective study of 12 months romosozumab followed by 12 months denosumab in long-term dialysis patients (dialysis vintage 67.0 months [23.5, 125.5], median PTH 80.0 pg/mL [39.0, 217.5], on calcimimetics 30.8%), no new fractures were noted [152].

*BMD:* The study by Sato et al. cited above (n = 131) found significant and large 6- and 12-months BMD increases with romosozumab at the lumbar spine (8.7% [9.8] and 15.3% [12.9]) and the femoral neck (3.1% [7.2] and 7.2% [8.3]), respectively. Controls did not show any BMD changes at either site during the study period [151]. Teraguchi et al. compared romosozumab (n = 21) to densoumab (n = 24) and found significantly superior BMD gains at the lumbar spine (14.6% [9.2] vs. 6.3% [7.8]; *p* < 0.05), but not at the femoral neck (4.3% [7.7] vs. 6.0% [6.5]; *p* = 0.41) [153]. Saito et al. investigated 12 month romosozumab followed by 12 month denosumab treatment in 13 patients (cited above). They found significant BMD changes after 12 months romosozumab only at the lumbar spine (BMD 9.0% [16.0, 5.0]; *p* < 0.001, T-scores −1.1 [−0.4, −2.0] to −0.4 [0.8, −1.0]; *p* < 0.001). However, after additional 12 months under denosumab treatment, BMD increases were significant at all sites (LS: 14.9% [18.8, 8.8]; *p* < 0.01, FN 6.5% [9.8, 0.4]; *p* < 0.05, TH 5.4% [14.6, −0.4]; *p* < 0.05) [152].

**Kidney transplant:**

Up to date, there is hardly any data on the effect of romosozumab in KTRs.

*Fractures:* A very small retrospective case series investigated KTRs with osteoporosis (n = 12, n = 8 with history of fractures, 83% females) and a mean eGFR of 38.2 (2.8) ml/min/1.73 m^2^, receiving romosozumab as first-line treatment (n = 8) or transitioning from bisphosphonates (n = 3) or teriparatide (n = 1). Within the 12-months follow-up, no further fractures were noted [154].

*BMD:* In the small case series cited above, average BMD changes from baseline to 6 and 12 months were considerable (LS: 10.11% (2.57) and 15.18% (4.09), FN 3.27% (2.14)% and 6.34% (3.77), TH 3.79% (1.22) and 8.83% (2.36) [154].

### 9.3. Safety of Romosozumab

Romosozumab is generally well tolerated. Across CKD, dialysis, and KTR populations, no study has demonstrated increased incidence of CV mortality, severe hypocalcemia (under substitution), osteonecrosis of the jaw, or atypical femoral fractures with romosozumab.

*Non-CKD:* In the large non-CKD population investigations, however, a slight increase in selected CV events has been reported, which has been a matter of debate since. In the ARCH trial (n = 4093), cardiac ischemic events occurred in 16 (0.8%) patients with romosozumab versus in 6 (0.3%) patients receiving placebo (OR 2.65 [95% CI, 1.03–6.77]) [155]. In the BRIDGE trial (n = 245), CV SAEs were reported in eight (4.9%) versus two (2.5%) patients [156]. An FDA pharmacovigilance analysis found an OR of 4.07 (95% CI, 2.39–6.93) for major CV events and a meta-analysis of RCTs reported a RR for four-point major adverse cardiovascular events (4P MACE) of 1.39 (95% CI, 1.01–1.90; *p* = 0.04) comparing romosozumab with placebo [157]. However, the largest RCT evaluating romosozumab versus placebo (FRAME, n = 7180) did not identify an elevated CV risk [158] and several contextual factors mitigate concern regarding CV safety signals of cited studies: In ARCH, the comparator was a bisphosphonate, which have been associated, though inconsistently, with CV protection. During the 2-years trial extension, the incidence of CV events in the alendronate arm rose to approximate that of the romosozumab alendronate arm, suggesting that the initially low event rate in the alendronate group may have been a chance effect. In BRIDGE, fewer participants in the romosozumab compared with the placebo group received cardioprotective therapies despite a history of CV disease. In the FDA pharmacovigilance study, the observed safety signal was driven largely by Japanese patients, who, next to those experiencing a CV event, displayed a higher CV risk profile (older age, more male patients, and less frequent use of cardioprotective medication). In the meta-analysis, 4P-MACE were not increased when comparing romosozumab not to all other therapies combined but with placebo or alendronate alone. Inclusion of RCTs without CV events also abrogated statistical significance. In addition, no increase was observed in other CV outcomes including total CV events, 3P-MACE, myocardial infarction, CV mortality, stroke, heart failure, atrial fibrillation, hypertension, or aneurysmal disease (aortic or intracranial).

*CKD:* Post hoc analyses of FRAME and ARCH revealed no differences in adverse events by baseline kidney function. In patients with eGFR 30–59 mL/min/1.73 m^2^, adjudicated CV event rates were 12 (1.6%) versus 10 (1.6%) for romosozumab versus placebo in FRAME, and 14 (2.6%) versus 8 (1.7%) for romosozumab versus alendronate in ARCH. Notably, no hypocalcemia events were reported in this subgroup under either treatment [150].

*Dialysis:* In the largest prospective controlled cohort of dialysis patients (n = 131), as well as in a smaller prospective study with denosumab as comparator (n = 25), romosozumab did not increase the incidence of new CV events during one year of treatment (5.2% vs. 10.9% in controls; no events in the denosumab study) [151,153]. A very small uncontrolled cohort (n = 13) reported new CV events in two patients, and demonstrated significant increases in coronary artery and thoracic aorta calcification scores after 12 months of treatment (CACS: 676.2 [2497.9, 71.3] to 1208.0 [2558.8, 105.6]; *p* = 0.045. TACS: 11,186.6 [27,224.2, 4294.8] to 15,383.2 [36,684.2, 6782.6]; *p* = 0.002) [152]. With respect to hypocalcemia, both Sato et al. and Saito et al. observed similar declines in corrected serum calcium from baseline (9.5 ± 0.8 and 9.6 [10.0, 8.9] mg/dL, respectively) to nadirs at 6 weeks (8.9 ± 0.7 and 8.8 [9.3, 8.4] mg/dL, respectively), followed by partial recovery by 12 months, though still below baseline (*p* < 0.0001). These changes occurred despite concurrent calcium and/or vitamin D supplementation regimens, which were protocolized and titrated according to serum calcium concentrations (Sato et al.: i.v. maxacalcitol [active d] 2.5 [2.7] up to 3.8 [3.1] mg/week or p.o. 1-calcidiol [pro-hormone] 0.11 [0.19] up to 0.17 [0.59] g/day. Saito et al.: 1–1.5 g elemental calcium/day [ca-carbonate] plus alfacalcidol 0.25–0.5 μg/day with regular adaptations if calcium < 8.5 mg/dL [increase in ca-carbonate by 500 mg/day) or <8.0 mg/dL [increase in alfacalcidol 0.25 μg/day]) [151,152].

*Kidney Transplant:* In a one-year case series (n = 12), no CV events or episodes of hypocalcemia were observed [154].

### 9.4. Pragmatic Clinical Approach

For patients with advanced CKD, the evidence base remains very limited and excludes individuals with an eGFR < 30 mL/min/1.73 m^2^. Nonetheless, in patients with CKD stages 1–3, the available data are of considerable scientific value and demonstrate a substantial improvement of fracture incidence and BMD. For dialysis patients, limited evidence from Japanese cohorts shows increases in BMD but no fracture risk reduction. For KTRs, hardly any evidence is available. A small case series might suggest a stabilization of prior fracture occurrence along with substantial BMD gains.

Romosozumab is generally well tolerated. Across CKD cohorts (including patients on dialysis and KTRs), no study to date has reported an increased incidence of CV mortality, severe hypocalcemia under appropriate supplementation, osteonecrosis of the jaw, or atypical femoral fractures. The relevance of the CV safety concerns is a matter of debate (see evidence and discussion above). A history of myocardial infarction or stroke constitutes a contraindication although both clinical practice and available studies often limit this consideration to a one-year period preceding planned romosozumab initiation. Hypocalcemia is mostly moderate but should be monitored for and temporary substitution of calcium and vitamin D should be considered (preemptive substitution in the context of extended surveillance intervals).

Romosozumab therapy is typically limited to a one-year course. Although, in contrast to denosumab, cessation is not followed by a rebound in fracture risk, sequential antiresorptive treatment remains advisable to sustain and consolidate osteoanabolic benefits. The osteoanabolic properties of romosozumab suggest potential benefits also in the context of ABD. Dosing is generally independent of kidney function.

## 10. Teriparatide

### 10.1. Rationale and Guidelines

Teriparatide is a recombinant PTH (1–34) analogue, established as an osteoanabolic treatment in osteoporosis with very high fracture risk. In CKD, there is limited evidence predominantly for ABD, where risks for bone and CV health is increased and treatment options sparce. Within nephrology guidelines, teriparatide is mentioned only marginally, with reference to potential benefits in low turnover bone disease and possible adverse effects such as hypercalcemia.

### 10.2. Evidence on Fracture Prevention and Bone Mineral Density

**Advanced CKD:**

Fractures: Evidence is very scarce. A post-marketing surveillance analysis comprising patients with CKD stage 4 (n = 30) and stage 5 (n = 3) found only one fracture incidence during 24 months of teriparatide. A total of 82% of participants had suffered a fracture before treatment with teriparatide [159]. In a cardiac transplant patient with CKD (eGFR 21 mL/min/1.73 m^2^) and multiple osteoporotic fractures (10 vertebral, 3 peripheral), 24 months’ treatment with teriparatide was associated with no further fractures and improvements of static and dynamic parameters on bone histology [160].

BMD: In the post-marketing analysis cited above, patients with stage 4 CKD for whom BL and follow-up data was available (n = 6), BMD increased in all but one patient, but mean changes were not significant (22.2% [95% CI, −14.4 to 58.8]) [159]. In a non-controlled single-center prospective cohort (n = 71) comprising non dialysis (PTH < 150 pg/mL) and dialysis (PTH < 65 pg/mL) patients, BMD (g/cm^2^) increased significantly during 24 months of teriparatide treatment (mean [SEM] difference 0–3 and 3–6 months: lumbar spine: 0.142 [0.037]; *p* = 0.0002 and 0.113 [0.038]; *p* = 0.0038. Femoral neck: 0.120 [0.033]; *p* = 0.0005 and 0.170 [0.033]; *p* < 0.0001) [161]. In a long-term bisphosphonate user (20 years) with histologically proven ABD, an atypical femoral fracture and a eGFR of 51 mL/min/1.73 m^2^, 2 years’ treatment with teriparatide was associated with a femoral neck BMD improvement of 25.5% and improvements in bone histology (bone volume and bone formation) [162].

**Dialysis:**

Fractures: Data on fracture prevention is missing. In a patient with ABD and multiple fractures, no new fractures appeared during 24 months teriparatide treatment period. Two case reports with biopsy-proven ABD showed improvements in static and dynamic parameters of bone formation after 24 months [163] as well as high turnover and normal mineralization and bone volume after 9 months [164] of teriparatide treatment, respectively.

BMD: Evidence comprises a small RCT (n = 61) and three small prospective cohorts (n = 15, n = 22, n = 7). The RCT found significant 12-months BMD gains with teriparatide compared to controls (5.7 vs. −10.7 g/cm^3^) in patients without high bone turnover [165]. Observational evidence reports lumbar spine BMD gains in patients with low PTH or ABD of 2.5% to 3.4% (6 to 12 months, 20 μg/d), 3.3 (1.9)% to 3.0 (1.8)% (6 to 12 months, 56.5 μg/week) [166], and increases from 0.885 (0.08) to 0.914 (0.09) g/cm^2^; *p* < 0.02 (6 months, 20 μg/d) [167].

**Kidney transplant:**

Fractures: Evidence is very scarce. A retrospective cohort comparing teriparatide to alendronate in 153 KTRs with osteoporosis found no significant difference in fracture incidence (1 vs. 5; *p* > 0.05) [168]. A 6-months biopsy-based double-blind RCT (n = 26) found no improvements of histomorphometry or mineralization, possibly due to persistent dialysis-associated PTH resistance [169].

BMD: Whereas the single available RCT (n = 26) did not find improvements of BMD with teriparatide compared to placebo, two cohort studies report significant gains. Qui et al. compared the effect of teriparatide to alendronate and found 1 year BMD differences of 0.823 (0.081) vs. 0.787 (0.084); *p* < 0.05 (lumbar spine), 0.794 (0.077) vs. 0.762 (0.075); *p* < 0.001 (hip) and 0.805 (0.080) vs. 0.763 (0.084); *p* < 0.001, respectively [168]. Vetrano et al. investigated 18 KTRs with osteoporotic fractures and low bone turnover and found 2 years’ adjusted BMD gains from 0.941 (0.152) to 1.074 (0.154); *p* = 0.03 (lumbar spine) and from 0.753 (0.145) to 0.864 (0.141); *p* = 0.04 (total hip) with teriparatide [170].

### 10.3. Safety

Teriparatide is generally well tolerated. However, side effects do occur and tend to be significantly more frequent compared to bisphosphonates. Safety monitoring should comprise the risk of hypercalcemia, as well as reaction at the injection side, body pains, gastrointestinal symptoms, fatigue, hyperuricemia, calciphylaxis, and hypotension.

### 10.4. Pragmatic Clinical Approach

The evidence base for teriparatide in the CKD setting is very limited, with few controlled or robust studies available. Existing data suggest that teriparatide represents a feasible and reasonably safe option for osteoanabolic therapy in low bone-turnover situations or ABD. While treatment effects remain uncertain, there is some indication of potential benefits on bone turnover, BMD, and bone histology. In non-low-turnover bone disease, discontinuation of teriparatide should be followed by administration of antiresorptive therapy to prevent potential rapid bone loss. The tolerability of teriparatide is generally good. However, side effects are not uncommon and should be monitored.

## 11. Cost-Effectiveness

Key factors influencing cost-effectiveness include individual fracture risk; drug effect on fracture risk reduction; drug costs including route of administration, treatment duration, and monitoring requirements; necessary concomitant and follow-up therapies; complication rates; and patient adherence. US Medicare data suggest that bisphosphonates are substantially less expensive than other pharmacological classes [171]. According to estimates by the American College of Physicians, per-patient costs increase in the following order: bisphosphonates, romosozumab, denosumab, and teriparatide [171]. The authors concluded that bisphosphonates offer the most favorable balance among benefits, harms, patient values and preferences, and costs across available drug classes. A recent systematic review of cost-effectiveness analyses, encompassing 27 studies from 15 countries, found that 8 of 12 studies comparing traditional oral bisphosphonates with intravenous bisphosphonates, denosumab, or teriparatide demonstrated general cost-effectiveness or even dominance of these newer and more expensive agents [172]. In postmenopausal women with very high fracture risk, a sequential regimen of romosozumab (1 year)/denosumab (4 years) provided greater health benefits at lower cost compared with teriparatide (2 years)/denosumab (3 years) [173]. For sequential therapies, superior cost-effectiveness compared to monotherapy is hypothesized; however, additional clinical and economic data are required to substantiate this assumption [172]. We are not aware of cost-effectiveness analyses that have been published on pharmacological fracture prevention in patients with CKD.

## 12. Limitation

Despite a thorough review of the literature, this work does not claim to represent a systematic review. Thus, evidence base might be incomplete. The evidence presented pertains exclusively to fracture and BMD outcomes; other indications for the medications discussed should be evaluated separately.

## 13. Outlook

To resolve the decades-long therapeutic dilemma of medical fracture care in CKD, driven by invasive diagnostics, limited therapeutic options, scarce or inconsistent study results, and lack of RCT data, four steps seem essential. First, acknowledge the overriding fracture risk of CKD patients with its associated lethality and deterioration of autonomy and quality of life. Second, find confidence in reinstating bone biopsy as the only definitive diagnostic modality on a regular basis. Third, use available evidence to take pragmatic diagnostic and therapeutic decisions despite the absence of certainty, and last, seize the genuine opportunity to contribute meaningful knowledge to the community by publishing your experiences—no matter if limited to observational data or case reports.

## Figures and Tables

**Table 1:** The concept of chronic kidney disease–mineral and bone disorder (CKD-MBD).

Chronic Kidney Disease–Mineral and Bone Disorder (CKD-MBD)			
**Reduced Mineralization**	**More CKD-Specific Bone Disorder**		**Reduced Mass**
Esp. vitamin D deficiency Hypocalcemia Hypophosphatemia Aluminum toxicity Acidosis/RTA	2°/3° hyperparathyroidism Vitamin D deficiency Hypocalcemia Hyperphosphatemia Excess FGF23 Acidosis Uremia		Age Female gender Postmenopausal state Family history Steroids Renal Osteodystrophy Osteomalacia etc.
**Osteomalacia**	**Renal Osteodystrophy**		**Osteopenia/Osteoporosis**
	**Low turnover** Adynamic Bone Disease	**High turnover** Osteitis Fibrosa	

Note: All entities of CKD–MBD may lead to reduced bone mass, rendering them indistinguishable from one another on dual-energy X-ray absorptiometry (DEXA). The clinical course and markers of bone metabolism may provide clues to the predominant bone disease phenotype. However, only histologic assessment permits determination of the type and extent of the underlying pathophysiological processes.

**Table 2:** Blood values and cut-offs that may support a distinction between high turnover (osteitis fibrosa) and low turnover (adynamic bone disease) osteodystrophy.

		Low Turnover Adynamic Bone Disease		High Turnover Osteitis Fibrosa
	Reference Range			
*KDIGO 2017* [2] *(dialysis)*		**<2**-**times** ULN	**iPTH**	**>9-times** ULN
*Jorgensen* [3] *(CKD 4–5D, KTRs)*		**1.3-times** ULN [0.9–2.2]		**6.2-times** ULN [2.9–10.7]
*Jorgensen (CKD 4–5D, KTRs)*		**53.2** [36.8, 86.5]		**249** [114.5, 428.6] (ULN 40 pg/mL)
*Jorgensen (CKD 4–5D, KTRs)*		**<90.5** pg/mL (sens 69%, spez 52%)		**>143.5** pg/mL (sens 70%, spez 74%)
*Salam* [4] *(CKD 4–5D)*		**≤183** pg/mL (sens 70%, spez 53%)		**>327** pg/mL (sens 53%, spez 96%)
*Salam (CKD 4–5D)*		**172** (119–292) pg/mL		**347** (161–381) pg/mL
*Sprague* [5] *(dialysis)*		**68.2** [23.2–186.3] pg/mL		**382.6** [139.5, 865.5] pg/mL
*Sprague (dialysis)*		**<103.8** pg/mL		**>323** pg/mL
*Sprague (dialysis)*		**<2-times** ULN (sens 65%, spez 67%)		**>9-times** ULN (sens 37%, spez 86%)
*Sprague (dialysis)*		**<150** pg/mL (sens 68%, spez 61%)		**>300** pg/mL (sens 58%, spez 78%)
*KDOQI* [6]		**<150** pg/mL		**>300–600** pg/mL
*Hercz* [7] *(dialysis)*		**77** (61) pg/mL		**369** (32) pg/mL
*Up to date (dialysis)*		**<100** pg/mL		**>450** pg/mL
*Literature*		Often **<300** ng/mL		Often **>600** ng/mL
*Literature*		**normal/decreasing/low**	**AP**	**normal/increasing/high**
*Jorgensen (CKD 4–5D, KTRs)*	35–130 U/L	**63.0** [47.2, 86.8] U/L		**125.0** [101.0, 182.1] U/L
*Jorgensen (CKD 4–5D, KTRs)*	35–130 U/L	**<87** U/L (sens 64%, spez 57%)		**>97** U/L (sens 76%, spez 77%)
*Salam (CKD 4–5D)*		**≤88** IU/L (sens 91%, spez 63%)		**>102** IU/L (sens 65%, spez 73%)
*Salam (CKD 4–5D)*		**82** (53–86) U/L		**115** (82–156) IU/L
*Literature in general*		**normal/decreasing/low**	**Bone-specific AP**	**normal/increasing/high**
*Jorgensen (CKD 4–5D, KTRs)*	6.1–25.5 μg/L	**<24.2** (sens 87%, spez 58%)		**>33.7** (sens 73%, spez 86%)
*Jorgensen (CKD 4–5D, KTRs)*	6.1–25.5 μg/L	**15.3** [11.1, 22.1] µg/L		**47.4** [33.8, 66.8] µg/L
*Salam (CKD 4–5D)*		**17.7** (5.6) µg/L		**34.4** (13.3) µg/L
*Salam (CKD 4–5D)*		**≤21** μg/L (sens 89%, spez 77%)		**>31** μg/L (sens 56%, spez 83%)
*Sprague (dialysis)*	11.6–42.7 U/L	**28.2** [18.0, 46.2] U/L		**63.3** [42.3, 116.8] U/L
*Sprague (dialysis)*	11.6–42.7 U/L	**<33.1** U/L		**>42.1** U/L
*Up to date 2025 (CKD and dialysis)*				**≥20** ng/mL virtually excl. ABD, esp. if **PTH >200** pg/mL
*Sprague (dialysis)*	13.9–85.5 ng/mL	**348.3** [183.1, 599.6] ng/mL	**Total P1NP**	**787.0** [523.7, 992.2] ng/mL
*Sprague (dialysis)*	13.9–85.5 ng/mL	**<498.9** ng/mL		**>621.1** ng/mL
*Salam (CKD, dialysis, stage 4–5D)*		**≤124** ng/mL (sens 80%, spez 68%)		**>142** ng/mL (sens 75%, spez 68%)
*Salam (CKD 4–5D)*		**76.3** (51.7, 159.3) ng/mL		**214** (110.6–403) ng/mL
*Jorgensen (CKD 4–5D, KTRs)*	12.8–82.6 ng/mL	**<49.8** ng/mL (sens 80%, spez 70%)	**Intact P1NP**	**>120.7** ng/mL (sens 73%, spez 94%)
*Jorgensen (CKD 4–5D, KTRs)*	12.8–82.6 ng/mL	**31.5** [23.1, 44.7] ng/mL		**154.7** [119.0, 219.8] ng/mL
*Salam (CKD 4–5D)*		**≤57** ng/mL (sens 80%, spez 75%)		**>107** ng/mL (sens 53%, spez 93%)
*Salam (CKD 4–5D)*		**44.1** (29.2–68.4) ng/mL		**107.9** (63.5–182) ng/mL
*Jorgensen (CKD 4–5D, KTRs)*	1.1–6.9 U/L	**<3.44 U/L** (sens 73%, spez 74%)	**TRAP5b**	**>5.05** U/L (sens 77%, spez 76%)
*Jorgensen (CKD 4–5D, KTRs)*	1.1–6.9 U/L	**2.7** [1.9, 3.4] U/L		**6.4** [5.1, 8.5] U/L
*Salam (CKD 4–5D)*		**≤4.6** U/L (sens 89%, spez 71%)		**>4.6** U/L (sens 81%, spez 58%)
*Salam (CKD 4–5D)*		**3.2** (2.9–4.3) U/L		**5.8** (4.8–8.5) U/L
*Literature*		**Both higher**	**Calcium** **Phosphate**	**Calcium lower, Phosphate higher**

iPTH, intact parathyroid hormone; AP, total alkaline phosphatase; bone-specific AP, bone-specific alkaline phosphatase; PINP, procollagen type 1 N-terminal propeptide; TRAP5b, tartrate-resistant acid phosphatase 5b. Note: Bone-specific AP, intact P1NP, and TRAP5b are independent from renal clearance. Total P1NP is dependent on renal clearance. Bone-specific AP and intact P1NP are partly removed by hemodialysis. TRAP5b is not removed by dialysis. Clear distinction between high and low turnover osteodystrophy only via bone biopsy.

## Data Availability

No new data were created or analyzed in this study.
